# Supplementary material for: Rate-Limiting Steps in Yeast Protein Translation
Source: Cell. 2013 Jun 20;153(7):1589–601. doi: 10.1016/j.cell.2013.05.049 (PMC3694300; doi:10.1016/j.cell.2013.05.049)
Supplement: Data S2. Pseudocode for Whole-Cell Simulation of Protein Translation, Related to Figure 1 [file mmc2.pdf]

Pseudocode for whole-cell simulation of translation

Data:
  Genes:
    Total number of genes $= n$
  Ribosomes:
    Total number of ribosomes $= R^t$
  mRNAs:
    Number of mRNAs of gene $i = A_i$
  tRNAs:
    Number of tRNAs of type $\phi(j) = T^t_{\phi(j)}$
    Total number of tRNAs $\sum T^t_{\phi(j)} = T^t$
  List of other parameters
    Characteristic time of ribosomes $= \tau_r$
    Characteristic time of tRNAs $= \tau_t$
    Discrete positions a ribosome can occupy in a yeast cell $= N_r$
    Discrete positions a tRNA can occupy in a yeast cell $= N_t$
    Wobble parameter $= w$
    Scaling coefficient due to tRNA competition $= s$
    Also see Table S1
while *time* $< t$ *(total simulation time)* **do**
  Calculate
    Fraction of mRNAs of gene i that are *initiable*, f_i - i.e., those mRNAs with first 10 codons unbound.
    Number of *elongatable* ribosomes waiting at codon j, $R^b(j)$ - ribosomes with next 10 codons unbound.
    Rates of all possible events (see Table S2)
      Total initiation rate: $\rho^t = \sum_{i=1}^{n} \frac{R^f f_i A_i p_i}{\tau_r N_r}$
      Total elongation rate: $\epsilon^t = \sum_{j=1}^{61} \frac{R^b(j) T^f_{\phi(j)} w_j s}{\tau_t N_t}$
    Probability of each possible event (see Table S2)
  Randomly select an event based on its probability of occurrence (see Table S2)
  Update the changes in the state of the cell (see $\Delta State$ in Table S2)
  Increment *time* by $\frac{1}{\rho^t + \epsilon^t}$
  Update the number of free ribosomes, R^f
  Update the number of free tRNAs of type $\phi(j)$, $T^f_{\phi(j)}$
end
