# Supplementary material for: Rate-Limiting Steps in Yeast Protein Translation
Source: Cell. 2013 Jun 20;153(7):1589–601. doi: 10.1016/j.cell.2013.05.049 (PMC3694300; doi:10.1016/j.cell.2013.05.049)
Supplement: Document S1. Tables S1–S3 [file mmc3.pdf]

Table S1: Complete List of Model Parameters, Related to Figure 1 and Table 1

Para	Description	Value	References
R^t	Number of ribosomes	2×10^5	(von der Haar, 2008; Warner, 1999)
A^t	Number of mRNAs	6×10^4	(Zenklusen *et al.*, 2008)
T^t	Number of tRNAs	3.3×10^6	(Waldron & Lacroute, 1975)
T_n	Number of types of tRNAs	41	(Chan & Lowe, 2009)
T_j^t	Number of tRNAs of type j	$\sim 12,000 - 190,000$	(Chan & Lowe, 2009)
A_i	Number of mRNAs of gene i	$1 - 1254$	(Ingolia *et al.*, 2009)
p_i	Gene-specific initiation probability	$\sim 3.5 \times 10^{-6} - 0.115$	(Experimental Methods)
n	Number of genes	3795	(Ingolia *et al.*, 2009)
D_r	Diffusion coefficient of ribosomes	$3 \times 10^{-13} \mathrm{m}^2/\mathrm{s}$	(Politz *et al.*, 2003)
D_t	Diffusion coefficient of tRNAs	$8.42 \times 10^{-11} \mathrm{m}^2/\mathrm{s}$	(Werner, 2011)
C_r	Size of ribosome in codons	10	(Ingolia *et al.*, 2009)
s	tRNA competition coefficient	7.78×10^{-4}	(Experimental Methods)
V	Volume of the cell	4.2×10^{-17} m^3	(Jorgensen *et al.*, 2002)
R^b	Number of bound ribosomes		(Extended Experimental Procedure)
R^f	Number of free ribosomes		(Extended Experimental Procedure)
T_j^b	Number of bound tRNAs of type j		(Extended Experimental Procedure)
T_j^f	Number of free tRNAs of type j		(Extended Experimental Procedure)
ϵ_i	Total elongation rate of gene i		(Extended Experimental Procedure)
ρ_i	Initiation rate of gene i		(Extended Experimental Procedure)
L_i	Length of gene i in codons	$36 - 4910$	(Ingolia *et al.*, 2009)
$u_{j,i}$	Frequency of codon j in gene i		(Extended Experimental Procedure)
λ_r	Characteristic length of ribosomes	3×10^{-8} m	(Politz *et al.*, 2003)
λ_t	Characteristic length of tRNAs	1.5×10^{-8} m	(Nissen *et al.*, 1999)
τ_r	Characteristic time of ribosomes	5×10^{-4} s	(Extended Experimental Procedure)
τ_t	Characteristic time of tRNAs	4.45×10^{-7} s	(Extended Experimental Procedure)
N_r	Positions for ribosomes in volume V	1.56×10^6	(Extended Experimental Procedure)
N_t	Positions for tRNAs in volume V	1.24×10^7	(Extended Experimental Procedure)
t	Total simulation time	1,500 s	(Extended Experimental Procedure)
t_b	Burn-in simulation time	1,000 s	(Extended Experimental Procedure)
w	Wobble parameters	$w_{rr/yy} = 0.61$<br>$w_{ry/yr} = 0.64$	(Curran & Yarus, 1989; Lim & Curran, 2001)

Table S2. Markov States and Transition Rates, Related to Figure 1

Initiation	Gene #	mRNA # of gene			Initiation rate	Event probability	ΔState
	1	1			0	0	N/A
	1	2			0	0	N/A
	1	3			$\frac{R^f p_1}{\tau_r N_r}$	$\frac{R^f p_1}{\tau_r N_r(\rho^t+\epsilon^t)}$	$R^f \to R^f - 1$
	1	A_1			$\frac{R^f p_1}{\tau_r N_r}$	$\frac{R^f p_1}{\tau_r N_r(\rho^t+\epsilon^t)}$	$R^f \to R^f - 1$
	2	1			$\frac{R^f p_2}{\tau_r N_r}$	$\frac{R^f p_2}{\tau_r N_r(\rho^t+\epsilon^t)}$	$R^f \to R^f - 1$
	2	2			$\frac{R^f p_2}{\tau_r N_r}$	$\frac{R^f p_2}{\tau_r N_r(\rho^t+\epsilon^t)}$	$R^f \to R^f - 1$
	2	.	.	.	.	.	.
	2	A_2			0	0	N/A
	.	.	.	.	.	.	.
	n	.	.	.	.	.	.
	n	A_n			$\frac{R^f p_n}{\tau_r N_r}$	$\frac{R^f p_n}{\tau_r N_r(\rho^t+\epsilon^t)}$	$R^f \to R^f - 1$
				Total initiation rate	$\rho^t = \sum_{i=1}^{n} \frac{R^f f_i A_i p_i}{\tau_r N_r}$		
**Elongation**	**Gene #**	**mRNA # of gene**	**Codon position**	**Ribosome bound**	**Elongation rate**	**Event probability**	**ΔState**
	1	1	1	N	0	0	N/A
	1	1	2	Y	$\frac{T^f_{\phi(1,2)} w_{1,2} s}{\tau_t N_t}$	$\frac{T^f_{\phi(1,2)} w_{1,2} s}{\tau_t N_t(\rho^t+\epsilon^t)}$	Ribosome bound at codon 2 →N<br>Ribosome bound at codon 3 →Y
	1	1	3	N	0	0	N/A
	1	1	.	.	.	.	.
	1	1	L_1	N	0	0	N/A
	1	2	1	Y	0	0	N/A
	1	2	.	.	.	.	.
	1	2	11	Y	$\frac{T^f_{\phi(1,11)} w_{1,11} s}{\tau_t N_t}$	$\frac{T^f_{\phi(1,11)} w_{1,11} s}{\tau_t N_t(\rho^t+\epsilon^t)}$	Ribosome bound at codon 11 →N<br>Ribosome bound at codon 12 →Y
	1	2	.	.	.	.	.
	1	2	L_1	Y	$\frac{T^f_{\phi(1,L_1)} w_{1,L_1} s}{\tau_t N_t}$	$\frac{T^f_{\phi(1,L_1)} w_{1,L_1} s}{\tau_t N_t(\rho^t+\epsilon^t)}$	$R^f \to R^f + 1$
	1	.	.	.	.	.	.
	1	A_1	L_1	.	.	.	.
	2	.	.	.	.	.	.
	2	A_2	L_2	.	.	.	.
	.	.	.	.	.	.	.
	n	A_n	L_n	.	.	.	.
				Total elongation rate	$\epsilon^t = \sum_{j=1}^{61} \frac{R^b(j) T^f_{\phi(j)} w_j s}{\tau_t N_t}$		

A precise definition of the state space for our Markov model of translation. The table describes the state of the cell and all possible transitions to other states, along with their associated rates. The first column gives the gene number, which ranges from1 to *n* = 3,795 in our model. The second column indicates the mRNA number of each gene *i*, which ranges from 1 to A_i. The third column indicates the codon position in a given

mRNA molecule of gene *i*, which ranges from 1 to L_i. The fourth column indicates whether or not a ribosome is currently bound at each codon position in each mRNA of each gene. In the example illustrated here, the current state features a ribosome bound at gene 1 – mRNA 1 – codon 2, at gene 1 – mRNA 2 – codon 1, at gene 1 – mRNA 2 – codon 11, etc. The fifth column gives the initiation rate of each mRNA molecule, which depends upon the current number of free ribosomes, the characteristic time of a ribosome, the number of positions a ribosome can occupy in the cell, and the gene-specific initiation probability p_i. Furthermore, a gene cannot be initiated if any ribosome is bound within its first 10 codons. The fifth column also indicates the elongation rate of each codon in each mRNA, which depends upon the number of free cognate tRNAs, the wobble parameter, the tRNA competition coefficient *s*, the characteristic time of a tRNA, and the number of position a tRNA can occupy in the cell. Furthermore, elongation at a codon is not possible if another ribosome is bound to the same mRNA molecule within the subsequent 10 codons. The last column indicates the change in state that would be caused by the associated initiation or elongation event. For instance, upon an initiation event, the number of free ribosomes in the cell, R^f decreases by 1. Alternatively, if the ribosome bound at gene 1 – mRNA 1 – codon 2 undergoes elongation, then it frees the ribosome bound at position 2 and adds a ribosome bound at position 3. Finally, if the ribosome bound at gene 1 – mRNA 2 – codon L_1 undergoes elongation, then this leads to termination of translation and increases the number of free ribosomes in the cell, R^f, by 1.

Table S3. List of Ribosomal Genes Used for Estimating RSCU, Related to Figure 2

YBL027W	YBL072C	YBL087C	YBL092W
YBR031W	YBR048W	YBR084CA	YBR181C
YBR189W	YBR191W	YCR031C	YDL061C
YDL075W	YDL081C	YDL082W	YDL083C
YDL130W	YDL136W	YDL184C	YDL191W
YDR012W	YDR025W	YDR064W	YDR382W
YDR418W	YDR447C	YDR450W	YDR471W
YDR500C	YEL054C	YER056CA	YER074W
YER102W	YER117W	YER131W	YFL034CA
YFR031CA	YGL030W	YGL031C	YGL076C
YGL103W	YGL123W	YGL135W	YGL147C
YGL189C	YGR027C	YGR034W	YGR085C
YGR118W	YGR148C	YGR214W	YHL001W
YHL015W	YHL033C	YHR010W	YHR021C
YHR141C	YHR203C	YIL018W	YIL052C
YIL069C	YIL133C	YIL148W	YJL136C
YJL177W	YJL189W	YJL190C	YJL191W
YJR094WA	YJR123W	YJR145C	YKL006W
YKL156W	YKL180W	YKR057W	YKR094C
YLL045C	YLR029C	YLR048W	YLR061W
YLR075W	YLR167W	YLR185W	YLR264W
YLR287CA	YLR325C	YLR333C	YLR340W
YLR344W	YLR367W	YLR388W	YLR406C
YLR441C	YLR448W	YML024W	YML026C
YML063W	YML073C	YMR121C	YMR142C
YMR143W	YMR194W	YMR230W	YMR242C
YNL067W	YNL069C	YNL096C	YNL162W
YNL178W	YNL301C	YNL302C	YOL039W
YOL040C	YOL120C	YOL121C	YOL127W
YOR063W	YOR096W	YOR167C	YOR182C
YOR234C	YOR293W	YOR312C	YOR369C
YPL079W	YPL081W	YPL090C	YPL131W
YPL143W	YPL198W	YPL220W	YPR043W
YPR102C	YPR132W		
