# Supplementary material for: Rate-Limiting Steps in Yeast Protein Translation
Source: Cell. 2013 Jun 20;153(7):1589–601. doi: 10.1016/j.cell.2013.05.049 (PMC3694300; doi:10.1016/j.cell.2013.05.049)
Supplement: Document S2. Article plus Supplemental Information [file mmc6.pdf]

Cell

Rate-Limiting Steps in Yeast Protein Translation

Premal Shah,[1] Yang Ding,[1] Malwina Niemczyk,[2,3] Grzegorz Kudla,[2,4] and Joshua B. Plotkin[1,*]
[1]Department of Biology, University of Pennsylvania, Philadelphia, PA 19104, USA
[2]Wellcome Trust Centre for Cell Biology, University of Edinburgh, Edinburgh EH3 9LP, UK
[3]Present address: Cancer Research UK Cambridge Institute, University of Cambridge, Cambridge CB2 0RE, UK
[4]Present address: Institute of Genetics and Molecular Medicine, University of Edinburgh, Edinburgh EH4 2XU, UK
*Correspondence: jplotkin@sas.upenn.edu
http://dx.doi.org/10.1016/j.cell.2013.05.049

SUMMARY

Deep sequencing now provides detailed snapshots of ribosome occupancy on mRNAs. We leverage these data to parameterize a computational model of translation, keeping track of every ribosome, tRNA, and mRNA molecule in a yeast cell. We determine the parameter regimes in which fast initiation or high codon bias in a transgene increases protein yield and infer the initiation rates of endogenous *Saccharomyces cerevisiae* genes, which vary by several orders of magnitude and correlate with 5′ mRNA folding energies. Our model recapitulates the previously reported 5′-to-3′ ramp of decreasing ribosome densities, although our analysis shows that this ramp is caused by rapid initiation of short genes rather than slow codons at the start of transcripts. We conclude that protein production in healthy yeast cells is typically limited by the availability of free ribosomes, whereas protein production under periods of stress can sometimes be rescued by reducing initiation or elongation rates.

INTRODUCTION

Protein translation is central to cellular life. Although individual steps in translation such as the formation of the 43S preinitiation complex are known in intricate molecular detail, a global understanding of how these steps combine to set the pace of protein production for individual genes remains elusive (Jackson et al., 2010; Plotkin and Kudla, 2011). Factors such as biased codon usage, gene length, transcript abundance, and initiation rate are all known to modulate protein synthesis (Bulmer, 1991; Chamary et al., 2006; Cannarozzi et al., 2010; Tuller et al., 2010a; Shah and Gilchrist, 2011; Plotkin and Kudla, 2011; Gingold and Pilpel, 2011; Chu et al., 2011; Chu and von der Haar, 2012), but how they interact with one another to collectively determine translation rates of all transcripts in a cell is poorly understood. Systematic measurements for some of the most critical rates—such as the gene-specific rates of 5′ UTR scanning and start codon recognition—are extremely difficult to perform. As a result, questions as fundamental as the relative role of initiation versus elongation in setting the pace of protein production are still actively debated (Kudla et al., 2009; Tuller et al., 2010a; Plotkin and Kudla, 2011; Gingold and Pilpel, 2011; Chu et al., 2011; Chu and von der Haar, 2012; Ding et al., 2012). Biotechnical applications that exploit these processes stand to gain from a quantitative understanding of the global principles governing protein production (Gustafsson et al., 2004; Salis et al., 2009; Welch et al., 2009).

Recent advances in synthetic biology allow high-throughput studies on the determinants of protein production (Kudla et al., 2009; Welch et al., 2009; Salis et al., 2009). Sequencing techniques such as ribosomal profiling provide snapshots of the translational machinery in a cell (Ingolia et al., 2009; Reid and Nicchitta, 2012). One way to leverage this new information is to develop a computationally tractable model of translation in a cell, to parameterize it from known measurements, and to use it to infer any unknown parameters of global translation dynamics.

Here, we develop a whole-cell model of protein translation, and we apply it to study translation dynamics in yeast. Our model describes translation dynamics to the single-nucleotide resolution for the entire transcriptome. In combination with ribosomal profiling data, we use our model to infer the initiation rates of all abundant yeast transcripts. We systematically explore how the codon usage, transcript abundance, and initiation rate of a transgene jointly determine protein yield and cellular growth rate. Applied to the endogenous genome, our model reproduces one of the defining features of ribosomal profiling measurements: a decrease in ribosome density with codon position. We evaluate both elongation- and initiation-driven hypotheses for the ramp of 5′ ribosome densities. We also describe the factors that influence ribosomal pausing along mRNA molecules, as well as the effects of stress on translation.

RESULTS

Model

We developed a continuous-time, discrete-state Markov model of translation. The model tracks all ribosomes and transfer RNA (tRNA) molecules in a cell—each of which is either freely diffusing or bound to a specific messenger RNA (mRNA) molecule at a specific codon position at any time point (Extended Experimental Procedures). Rates of initiation and elongation

Table 1. Summary of Model Parameters

Parameter	Description	Value or Range of Values	References
R^t	number of ribosomes	2×10^5	(Warner, 1999; von der Haar, 2008)
A^t	number of mRNAs	6×10^4	(Zenklusen et al., 2008)
T^t	number of tRNAs	3.3×10^6	(Waldron and Lacroute, 1975)
T_n	number of types of tRNAs	41	(Chan and Lowe, 2009)
T^t_j	number of tRNAs of type j	~12,000–190,000	(Chan and Lowe, 2009)
A_i	number of mRNAs of type i	1–1,254	(Ingolia et al., 2009)
p_i	gene-specific initiation probability	$\sim 3.5 \times 10^{-6}$–0.115	(Experimental Procedures)
n	number of genes	3,795	(Ingolia et al., 2009)
D_r	diffusion coefficient of ribosomes	3×10^{-13} m^2/s	(Politz et al., 2003)
D_t	diffusion coefficient of tRNAs	8.42×10^{-11} m^2/s	(Werner, 2011)
C_r	size of ribosome footprint in codons	10	(Ingolia et al., 2009)
s	tRNA competition coefficient	7.78×10^{-4}	(Experimental Procedures)
V	volume of the cell	4.2×10^{-17} m^3	(Siwiak and Zielenkiewicz, 2010)

See also Table S1.

are based on physical parameters that have been experimentally determined in yeast, including the cell volume, the abundances of ribosomes and tRNAs, and their diffusion constants (Tables 1 and S1 available online). Transition rates among states are parameterized in seconds so that the model describes the dynamics of translation in real time, as opposed to using arbitrary discrete time steps. We provide a precise definition of the Markov state space, as well as pseudocode and complete source code in Data S1 and S2 and also Table S2.

Unlike many other models of translation (Gilchrist and Wagner, 2006; Mitarai et al., 2008; Reuveni et al., 2011), which treat each mRNA molecule in isolation and assume an inexhaustible supply of free ribosomes that initiate the message at a constant rate, our model keeps track of every tRNA, mRNA, and ribosome molecule in the cell simultaneously, and so it captures the indirect effects of one gene's translation on another's (Figure 1). In particular, if many ribosomes are engaged in translating the mRNAs of one gene, this reduces the pool of free ribosomes and tRNAs available to translate other genes.

Our model makes a number of simplifying assumptions. Most importantly, our model treats the total number of ribosomes, tRNA molecules, and mRNA molecules in the cell as fixed quantities because the dynamics of their production and decay are typically slower than those of protein translation (García-Martínez et al., 2004; Larson et al., 2011). We specify the total number of ribosomes and tRNA molecules to agree with their experimentally determined values in an exponential-phase yeast cell: 2×10^5 and 3.3×10^6, respectively (Waldron and Lacroute, 1975; Warner, 1999; von der Haar, 2008; Siwiak and Zielenkiewicz, 2010; Chu and von der Haar, 2012). We infer gene-specific initiation probabilities (Extended Experimental Procedures) so that 85% of ribosomes are bound to mRNAs in equilibrium in agreement with measurements in yeast (Arava et al., 2003; Zenklusen et al., 2008). We further assume that tRNA charging is fast, which is reasonable because 80% of all tRNAs are charged at any given time in exponential-phase cells (Varshney et al., 1991; Jakubowski and Goldman, 1992; Chu et al., 2011).

As a result of these parameters, the equilibrium number of free ribosomes available in the cell is typically smaller than the number of available charged tRNAs of each species. In this regime, we will show that protein production is generally limited by the rate of translation initiation in the sense that increasing the initiation probability of an mRNA molecule will typically increase the rate at which protein is produced, but increasing its codon elongation rates generally will not increase production. The initiation-limited regime agrees with the long-standing view of endogenous protein synthesis (Andersson and Kurland, 1990; Bulmer, 1991; Eyre-Walker and Bulmer, 1993; Lackner et al., 2007; Plotkin and Kudla, 2011), but it contrasts with other models of translation that assume an inexhaustible supply of ribosomes, which are always available for initiation of an mRNA regardless of how many ribosomes are bound to other mRNAs (Mitarai et al., 2008; Reuveni et al., 2011; Tuller et al., 2011).

We implemented our Markov model of translation using the Gillespie algorithm. We simulated 1,500 s of translation and extracted the final 500 s to collect data on translation dynamics in equilibrium (Experimental Procedures). Our implementation requires about 1,300 s of computation time to simulate all initiation and elongation events in a wild-type cell for 1,500 s. In these simulations, at equilibrium, the mean elongation rate is 9.3 aa/s (median = 9.5 aa/s), and the mean distance between consecutive bound ribosomes is 60 codons (median = 34). Both of these quantities agree with empirical measurements in yeast (Arava et al., 2003).

Codon Bias and Transgene Expression

Optimizing a transgene's codon usage to the tRNA content of a cell often improves protein yield (Gustafsson et al., 2004; Welch et al., 2009), but the underlying mechanisms have not been systematically explored. To study this in a quantitative model, we simulated translation of a transgene within the context of a *Saccharomyces cerevisiae* cell containing 3,795 endogenous genes whose transcript levels and gene-specific initiation

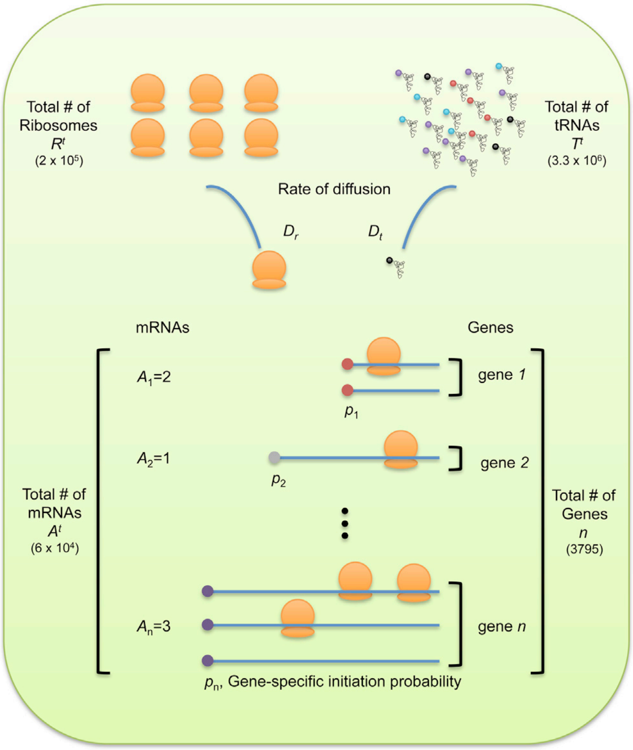

Figure 1. A Computational Model of Protein Translation

The model tracks the status of all ribosomes, tRNAs, and mRNAs in a cell in continuous time. At any time point, each tRNA and ribosome molecule is either diffusing freely in the cell or is bound to a specific mRNA molecule at a specific codon position. Translation initiation occurs when a free ribosome diffuses to an mRNA and subsequently, with an mRNA-dependent probability, scans to its start codon. The rate of elongation of each subsequent codon depends on the abundance of free cognate tRNAs and their diffusion to the bound ribosome. All rates are based on experimentally determined parameters, including the cell volume, numbers of mRNAs, total abundances of ribosomes and tRNAs, and their diffusion constants. A precise definition of the Markov state space, illustrative pseudocode, and the complete source code for simulation are provided in the Supplemental Information. See also Figure S1, Tables S1 and S2, and Data S1 and S2.

probabilities were estimated from ribosomal profiling data (Ingolia et al., 2009) (Experimental Procedures). By varying the codon adaptation index (CAI) (Sharp and Li, 1987) and transcript level of the transgene across many simulations, we delineated the regimes for which increasing codon bias is expected to increase protein yield and by what mechanisms.

Using the green fluorescent protein (GFP) as an example transgene, we found that increasing the CAI of a transgene significantly improves the rate of proteins produced per mRNA molecule only when the transgene mRNA accounts for a substantial proportion of all the mRNA in the transcriptome (Figure 2 and Table S4). For a transgene whose messages account for 50% of the cell's mRNA content, for example, increasing CAI from almost zero to one results in nearly 3.6-fold more proteins produced per transcript per second (Figure 2B, triangles), whereas optimizing CAI in a transgene expressed at only 1% of the transcriptome results in a more modest increase (~50%) in its rate of protein production (Figure 2B, squares). These results help explain the divergent views of biotechnological studies, which often report large gains in protein production upon optimizing transgene CAI (Gustafsson et al., 2004), and evolutionary studies of endogenous translation, which typically report very small effects of CAI on protein production per message (Bulmer, 1991; Tuller et al., 2010b; Gingold and Pilpel, 2011; Plotkin and Kudla, 2011). The discrepancy arises because transgenes are usually overexpressed and comprise a substantial fraction of all cellular mRNA, whereas endogenous genes are expressed at 1% of the transcriptome or less.

Why does codon bias strongly influence protein yield only when a gene has high mRNA abundance? The reason has to do with the effects of codon bias on the pool of free ribosomes, as seen in Figure 3. At equilibrium, neglecting rare abortion events, the rate of protein production from any given mRNA (i.e., the rate of polypeptide termination) must equal the rate of initiation on that mRNA, which, in turn, depends primarily on the abundance of free ribosomes in the cell. Increasing the CAI of a gene will increase its codon elongation rates and thus decrease the density of ribosomes on each of its mRNAs, but the overall effect on the pool of free ribosomes is small when the gene accounts for a small proportion (<1%) of mRNA in the transcriptome, as virtually all endogenous genes do. As a result, increasing the CAI of a gene at low mRNA abundance is not expected to strongly increase the rate of protein production, as our simulations confirm (Figure 2). By contrast, for a transgene at very high abundance (e.g., 50% of cellular mRNA), a significant fraction of all ribosomes in the cell are bound to its mRNAs. Increasing the CAI of such a gene leads to a significant increase in the pool of free ribosomes (Figure 3) and thus a significant increase in initiation rates and protein production from all mRNAs in the cell, including from the transgene itself.

Our simulations confirm the mechanistic role of free ribosomes in shaping the relationship between codon bias and protein yield. For a transgene at high abundance, such as 50% of the transcriptome, increasing its CAI causes a 3.2-fold increase in the equilibrium number of free ribosomes in the cell (Figure 3B), which accounts for the great majority of the concomitant 3.6-fold increase in its protein production. By contrast, for a transgene expressed at low levels (e.g., 1% of transcriptome), increasing CAI results in only 3% more free ribosomes (Figure 3B), which is not sufficient to explain the concomitant 50% increase in transgene protein production. (Nonetheless, a 3% fitness gain suffices to explain selection for codon bias in highly expressed endogenous genes over evolutionary timescales.) In this case, the gain in transprotein production is explained instead by reduced ribosomal trafficking at the 5′ end of transgene mRNAs: about 47% more transgene mRNAs are available to be initiated (that is, they are not bound by a ribosome at their 5′ end) when CAI ≈ 1 compared to CAI ≈ 0 in our simulations of such a transgene.

In summary, increasing transgene codon bias has a modest effect on translational efficiency, which is limited to the transgene mRNAs themselves and is caused by reduced ribosomal occupancy of their 5′ ends, whereas increasing CAI can have a huge effect on protein production globally—which is caused by an increased pool of free ribosomes—when the transgene

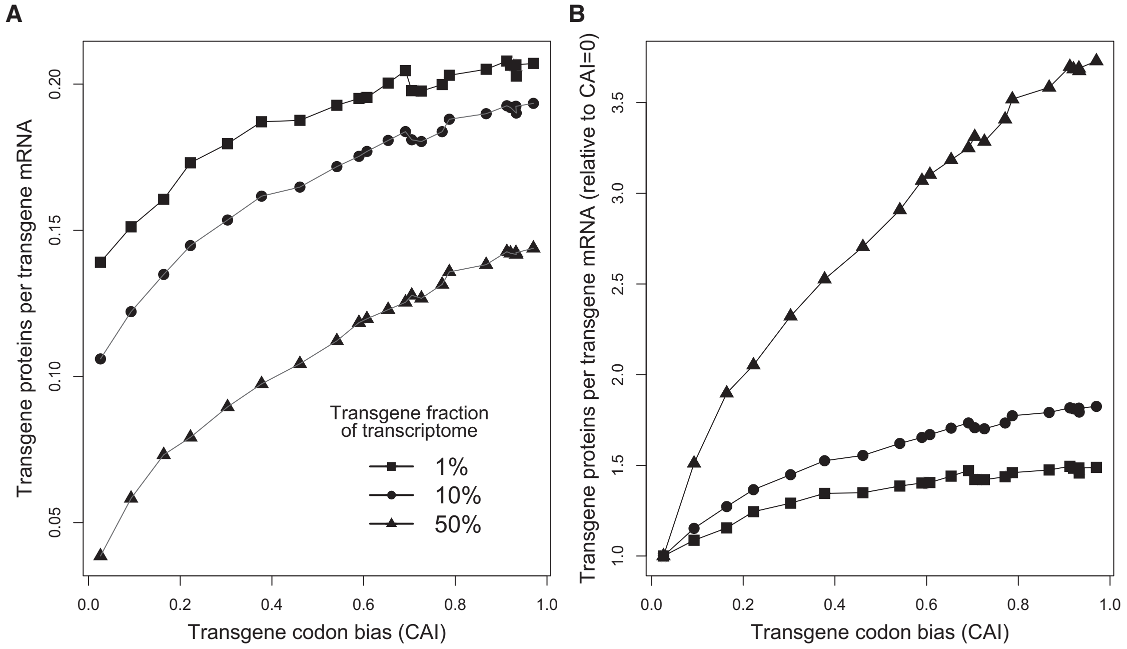

Figure 2. The Effects of Transgene Codon Bias on Protein Production

(A and B) We simulated translation in a wild-type yeast cell with the addition of a transgene. Transgene mRNA levels were set at 1%, 10%, or 50% of all cellular mRNA. We measured the number of transproteins produced per transgene mRNA over 500 s in equilibrium (A). As (A) shows, increasing the codon bias of the transgene generally increases the efficiency of its translation. However, when the transgene is expressed a low levels (e.g., transgene mRNAs constituting 1% of transcriptome), then the gain in translation efficiency achieved by optimizing codon bias is moderate (~50% gain, comparing CAI ≈ 1 to CAI ≈0, squares in [B]). By contrast, when the transgene mRNAs constitute a large fraction of the total transcriptome, then the gain in translation efficiency by optimizing codon bias is far greater (3.6-fold gain, triangles in [B]). See also Tables S3 and S4 and Figure S2.

has very high transcript abundance. These results (Figures 2 and 3) hold whenever protein translation is limited by the pool of ribosomes freely available for initiation, as is the case in healthy yeast cells (Arava et al., 2003; Zenklusen et al., 2008). When a cell is starved for tRNAs or amino acids, by contrast, or when the pool of available ribosomes is artificially inflated, the effects of codon bias on protein yield are due solely to reduced ribosomal interference along translating mRNAs, as discussed below.

Whereas Figures 2 and 3 quantify translation dynamics for a transgene expressed at three different abundances, Table S4 provides analogous results for a full range of transcript abundances. In the simulations described above, we maintained a constant transcriptome size in nucleotides so that an increase in the abundance of transgene mRNA comes at the expense of endogenous transcripts. Nonetheless, we found the same results when transgene mRNAs were simply added to the endogenous transcriptome (Table S4 and Experimental Procedures). Likewise, we found the same qualitative results for three other simulated transgenes with very different sequences and amino acid compositions than GFP (Table S4). Whereas Figure 2 reports the rate of protein production per transgene mRNA molecule, Table S4 reports the corresponding total rate of transprotein production in the cell, which is often the most important consideration in biotechnical applications. Most of the relationships between codon bias and protein yield per message also hold for total protein yield.

Initiation Rate and Transgene Expression

Translation initiation in eukaryotes is a multistep process involving multiple protein complexes. Our model simplifies this process into its two critical components: the arrival of a free ribosome at the 5′ end of an mRNA molecule, whose rate is determined by the number of free ribosomes and their diffusion constant, and the probability that such a ribosome then successfully binds and scans to the start site of the mRNA to irreversibly initiate translation. This initiation probability is known to depend strongly on the sequence of the transcript (Andersson and Kurland, 1990; de Smit and van Duin, 1990; Eyre-Walker and Bulmer, 1993; Kudla et al., 2009; Tuller et al., 2010b). In the simulations above, we set the initiation probability of the transgene at the 95th percentile of endogenous initiation probabilities because transgenes are typically optimized for rapid initiation (Salis et al., 2009; Welch et al., 2009). Here, we explore more generally how the probability of transgene initiation, once a ribosome has diffused to a transgene mRNA, influences protein production.

As Figure 4A shows, high codon bias will significantly increase protein yield only when the initiation probability of a transgene exceeds the (abundance-weighted) average initiation probability of the endogenous transcriptome. This is true irrespective of transgene abundance (Table S4), and it makes intuitive sense by considering, once again, the effects of initiation and elongation on the pool of free ribosomes. Increasing a gene's codon bias typically reduces the density of ribosomes along its mRNA

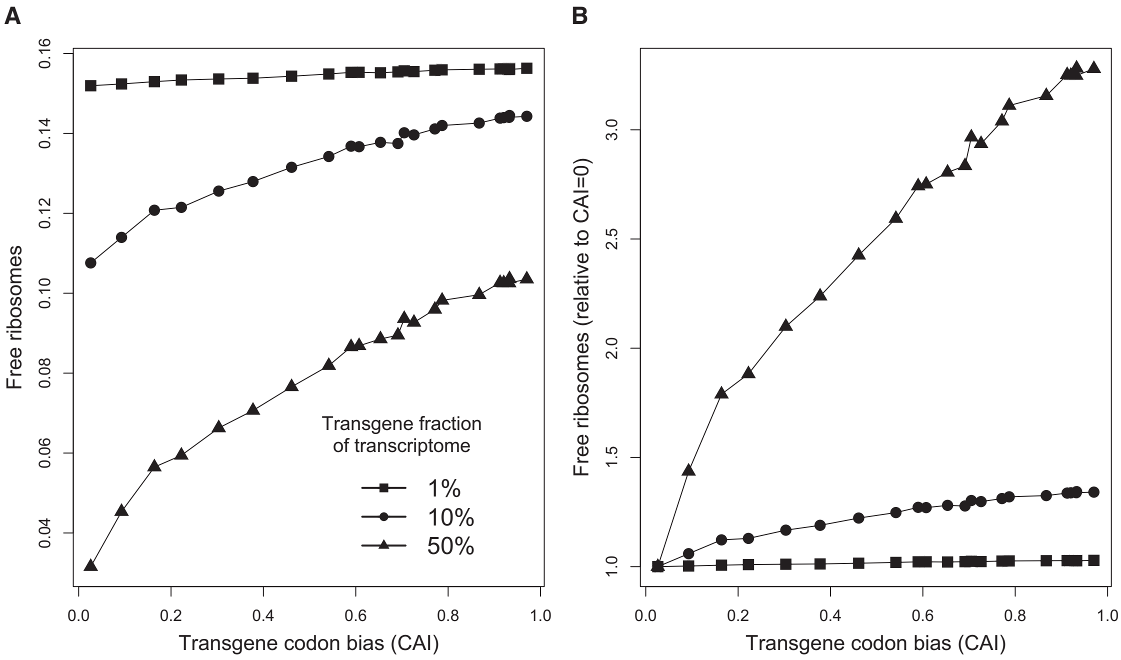

Figure 3. The Effects of Transgene Codon Bias on the Pool of Free Ribosomes

(A and B) As in Figure 2, we simulated translation of a transgene added to a wild-type yeast cell. Transgene mRNA levels were set at 1%, 10%, or 50% of all cellular mRNA. We measured the equilibrium fraction of ribosomes that are free (unbound). Increasing codon bias of the transgene reduces the number of ribosomes bound to its transcripts and thereby increases the pool of free ribosomes (A), especially when the transgene accounts for a large proportion of all cellular mRNA. For example, when transgene mRNAs comprise 50% of the total transcriptome, then optimizing codon bias of the transgene from CAI $\approx$ 0 to CAI $\approx$ 1 causes a 3.2-fold increase in the number of free ribosomes (triangles in [B]), which explains a large proportion of the corresponding gain in transprotein production. See also Table S4.

molecules due to faster elongation. When a highly expressed transgene has high initiation probability, its ribosomal density will be high as well, and so increasing codon bias can substantially replenish the pool of free ribosomes, which, in turn, increases initiation rates and protein yields. However, when a transgene has low initiation probability, regardless of its mRNA abundance, there are relatively few ribosomes bound to its mRNAs, and so increasing codon bias has a limited effect on its ribosomal densities and on the pool of free ribosomes (Figure 4B and Table S4). These results underscore the critical role of rapid initiation in allowing codon bias to modulate transgene protein yields.

Initiation Probabilities of Endogenous Genes

One of the most challenging problems in understanding protein translation remains the estimation of initiation rates for endogenous genes. As described above, translation initiation depends first on the arrival of a free ribosome to an mRNA and then on the ribosome binding and successfully scanning to the transcript's start codon (de Smit and van Duin, 1990). Despite their importance, the initiation probabilities of each transcript are the only parameters in our model that have not been measured empirically. Therefore, we used our model to infer the gene-specific initiation probabilities from ribosomal occupancy data (Ingolia et al., 2009).

To make this inference, we assumed that the cell is in equilibrium, and we derived analytic approximations for the steady-state density of ribosomes on each mRNA molecule (Extended Experimental Procedures) in terms of the unknown initiation probabilities. These approximations neglect the possibility of ribosomal interference along each message, but they are nonetheless extremely accurate in the parameter regime of a healthy yeast cell ($R > 0.9$; Figures S1A and S1B). We then inverted our equations to infer gene-specific initiation probabilities from observed densities of ribosomes on transcripts. An alternative method of estimating initiation probabilities from profiling data was independently developed by Siwiak and Zielenkiewicz (2010). We validated that our analytical method can indeed reliably infer initiation probabilities when we simulate ribosome profiling data for *S. cerevisiae* genes with known initiation probabilities (Figure S1B). Using this method, we inferred the initiation probabilities for the 3,795 *S. cerevisiae* genes whose ribosomal densities have been reliably measured (Ingolia et al., 2009).

The initiation probabilities we inferred for yeast genes vary by many orders of magnitude. According to these estimates, the average time between initiation events on a given mRNA molecule ranges from 4 s (fifth percentile) to 233 s (95th percentile), with a median value of 40 s. This variation provides the cell considerable range for tuning protein levels by modulating initiation probabilities of genes.

Experiments with individual genes (Hall et al., 1982; Duan et al., 2003) and with large sets of coding sequences (Kudla et al., 2009) suggest that strong 5′ mRNA structure reduces the rate of initiation, presumably by obstructing ribosomal-mRNA binding.

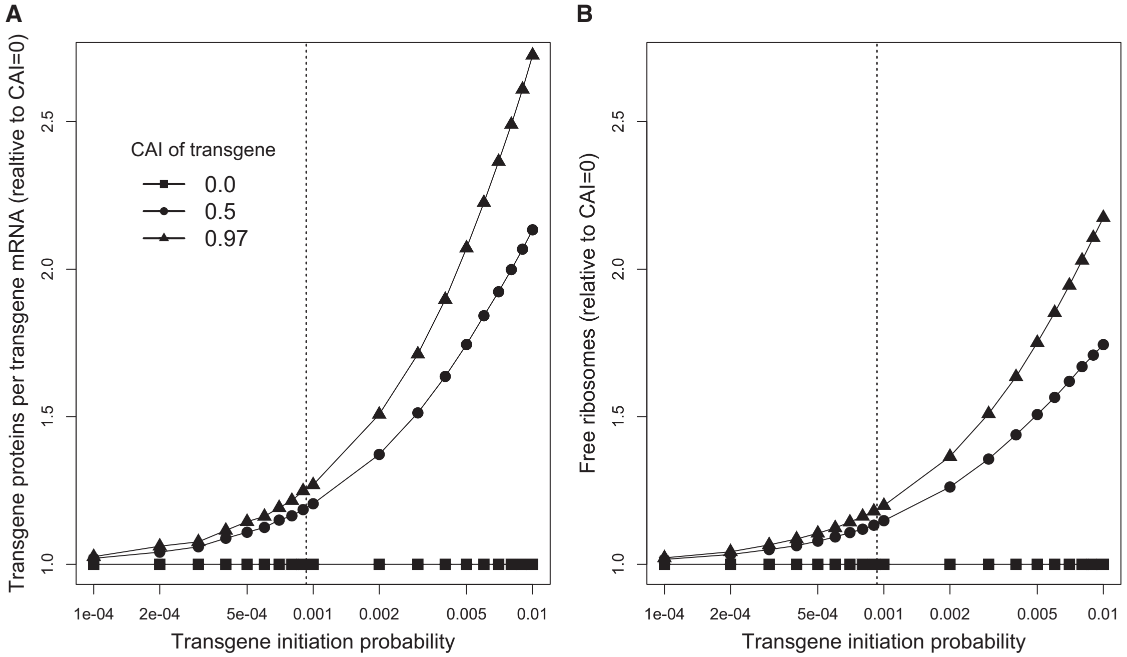

Figure 4. The Effects of Initiation Probabilities on Protein Production and Pool of Free Ribosomes

(A and B) As in Figure 2, we simulated translation of a transgene added to a wild-type yeast cell. Transgene mRNA levels were set at 25% of all cellular mRNA. We measured the number of transgene proteins produced per transgene mRNA (A), as well as the equilibrium fraction of ribosomes that are free (B); both quantities are expressed relative to the case of transgene with CAI $\approx$ 0. The dashed vertical line denotes the average initiation probability of endogenous yeast genes. Increasing codon bias of a transgene significantly increases the rate of protein production only when the transgene's initiation probability exceeds the average initiation probability of endogenous genes. See also Table S4 and Figure S2.

Using a large set of synthetic GFP genes that vary synonymously, we confirmed experimentally that 5′ mRNA folding plays a predominant role in determining protein levels in *S. cerevisiae* (Figure S2), which is similar to the role it plays in *Escherichia coli* (Kudla et al., 2009). In light of these experiments, we compared the initiation probabilities we estimated for 3,795 endogenous yeast genes with their predicted 5′ mRNA folding energies (nucleotides −4 to +37, Experimental Procedures) and found a strong positive correlation (Pearson correlation R = 0.125 and $p < 10^{-13}$; Figure 5A). These results suggest that 5′ mRNA structure systematically modulates initiation rates across the yeast genome.

Interestingly, we also found a negative correlation between initiation probability and open reading frame (ORF) length (R = −0.56 and $p < 10^{-15}$; Figure 5B), even after controlling for mRNA expression level (partial correlation, R = −0.425 and $p < 10^{-15}$). This trend suggests that shorter yeast genes have experienced selection for faster initiation, and so it provides a mechanistic explanation for the greater density of ribosomes typically observed on short genes (Arava et al., 2003; Lackner et al., 2007). Note that shorter genes are known to be more densely packed with ribosomes despite the fact that they tend to have significantly higher CAI (t test, $p < 10^{-4}$) and presumably faster elongation. This result again indicates the dominance of initiation, as opposed to elongation, in determining the density of ribosomes on transcripts.

We performed several controls to ensure that our estimates of initiation probabilities are not biased by gene length (Extended Experimental Procedures). We found no significant differences in the inferred initiation probabilities when artificially doubling the lengths of all transcripts (Kolmogorov-Smirnov, $p > 0.9$). Moreover, we validated that we can reliably infer initiation probabilities from simulated ribosomal profiling data even when gene length and initiation probabilities are positively correlated (Figures S1C and S1D and Extended Experimental Procedures), indicating that the negative correlation observed in the real yeast data is not an artifact of our inference procedure.

Why should short genes experience selection for fast initiation? Short genes are enriched for constitutively expressed housekeeping and ribosomal genes (Hurowitz and Brown, 2003), which must produce protein as rapidly as possible. In addition, housekeeping genes tend to have shorter 5′ UTRs and are under weaker posttranscriptional regulation (Hurowitz and Brown, 2003; Lin and Li, 2012). The probability of successful ribosomal binding and scanning on an mRNA may depend on the length of its 5′ UTRs; indeed, we find that genes with shorter 5′ UTRs exhibit higher inferred initiation probabilities ($p < 10^{-10}$). In addition to de novo initiation, recently terminated ribosomes can reinitiate translation on the same mRNA, a process known as ribosome recycling. The probability of successful reinitiation may depend on an mRNA's 3′ UTR length (Tanguay and Gallie, 1996; Gallie, 1998). Consistent with this hypothesis, we find genes with longer 3′ UTRs have higher initiation probabilities ($p < 10^{-5}$). However, unlike 5′ folding energy, we find no significant correlation between 3′ UTR folding energy and the initiation probability of a gene.

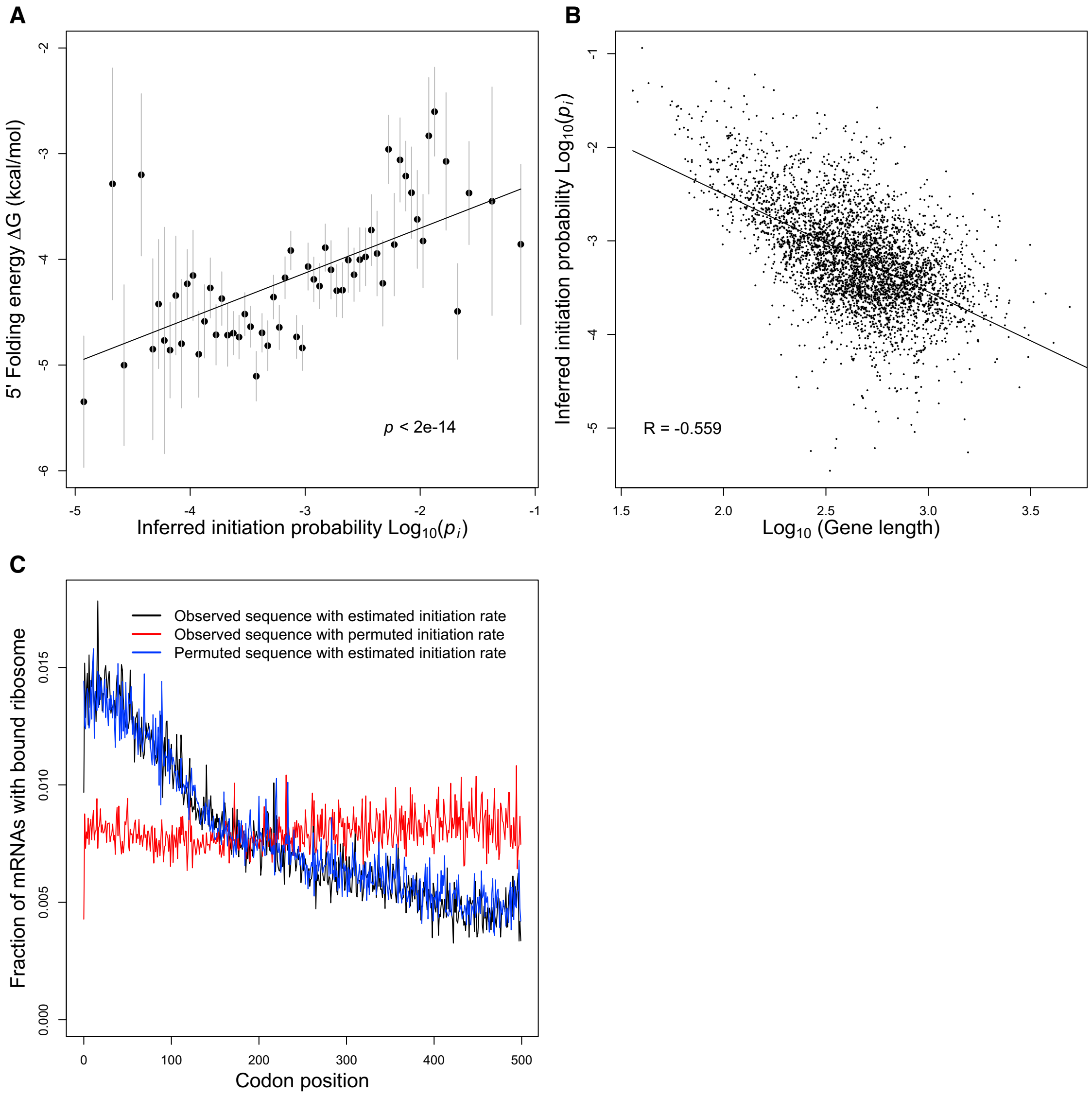

Figure 5. Fast Initiation of Short Genes Causes a 5′ Ribosomal Ramp

(A) Yeast genes with weak 5′ mRNA structure initiate quickly. We inferred the initiation probabilities of 3,795 endogenous yeast genes from ribosomal profiling data (Ingolia et al., 2009). A gene's initiation probability correlates strongly with the estimated energy of its 5′ mRNA structure. The gray bars indicate 1 SD of folding energies of binned genes.

(B) Initiation probabilities of yeast genes also correlate with ORF lengths, suggesting that short genes have experienced selection for faster initiation.

(C) Simulations of translation in a wild-type yeast cell recapitulate the “ramp” of 5′ ribosomes observed in empirical ribosomal profiling data (Ingolia et al., 2009). The figure shows the density of ribosomes bound to mRNAs as a function of codon position, averaged across the simulated transcriptome (black). The ramp is preserved in simulations that permute the codons within each gene (blue), but the ramp is disrupted when permuting the initiation probabilities among genes (red). Thus, we infer that the ramp of 5′ ribosomes is caused primarily by the trend toward faster initiation in short genes, rather than by the ordering of codons within each gene.

See also Figures S3, S4, and S5.

The “Ramp” of 5′ Ribosomes

A defining feature of ribosome profiling data in yeast (Ingolia et al., 2009) and humans (Reid and Nicchitta, 2012) is a striking decrease in ribosome density with codon position, averaged across the transcriptome. This observation has led to the “ramp” hypothesis, which attributes higher ribosome densities to slower codons in the 5′ ends of mRNAs (Tuller et al., 2010a; Reuveni et al., 2011; Tuller et al., 2011). Slow 5′ codons are thought to reduce ribosomal interference further down the length of the mRNA, leading to more efficient translation (Tuller et al., 2010a).

Our simulations of translation in a yeast cell recapitulate the empirical observation of declining ribosome density with codon position, averaged across the transcriptome (Figure 5C). The

ability of our model to recapitulate this striking spatial trend is nontrivial because we did not use any position-specific information from the ribosomal profiling data in order to parameterize the model (we used only the average ribosome density per mRNA).

Our computational model allows us to systematically determine which processes are responsible and which ones are dispensable in explaining the 5′-to-3′ ramp of decreasing ribosome density. We propose an alternate explanation for this trend: the ramp can be explained by the simple fact that shorter yeast genes tend to have higher initiation probabilities (Figure 5B) and correspondingly higher densities of ribosomes overall (Arava et al., 2003; Lackner et al., 2007). Because short genes are disproportionally weighted in early codon positions as opposed to late codon positions, their elevated ribosome densities will cause an apparent ramp in the transcriptome-wide average ribosome density with codon position.

We used our model to distinguish between our initiation-driven hypothesis and the elongation-driven hypothesis for the ramp of 5′ ribosomes (Tuller et al., 2010a, 2011; Reuveni et al., 2011). If the ramp were caused primarily by slow codons near the 5′ ends of genes, then the ramp would disappear upon randomizing codon order within each gene, whereas if the ramp were caused primarily by faster initiation rates in shorter genes, then it would disappear upon permuting initiation rates among genes. We found that simulations permuting codon order within genes still exhibit the ramp of 5′ ribosome densities (Figure 5C), whereas permuting initiation probabilities among genes removes the ramp (Figure 5C). Both of these results support the initiation-driven and reject the elongation-driven hypothesis for the cause of the 5′ ribosome ramp.

Aside from using our simulation model, we can also analyze the raw ribosomal profiling data of Ingolia et al. (2009) to dissect the causes of the apparent 5′ ribosome ramp. When we remove all positional information from the profiling data and use only the observed average ribosome density on each mRNA, assuming a uniform density along each mRNA, we still observe a decline in transcriptome-wide average ribosome density with codon position (Figure S3A). In addition, when inspecting the profiling data on a gene-by-gene basis, we find that just as many genes exhibit a trend of increasing ribosome density as exhibit a trend of decreasing ribosome density (Figure S3B and Extended Experimental Procedures). Finally, we have plotted average ribosome density by codon position for genes binned by ORF length, which is analogous to Figure S11 from Ingolia et al. (2009) but with more stringent length bins (Figure S4). These plots show no consistent 5′-to-3′ ramp, and many show 3′-to-5′ ramps (Figure S4). Taken together, these analyses of the raw profiling data confirm the conclusions drawn from our simulations: the apparent 5′ ribosome ramp in yeast is not caused primarily by a higher density of ribosomes near the 5′ end of each message but rather by a greater overall density of ribosomes on shorter mRNA molecules due to their faster rates of initiation.

Comparison to Other Models of Translation

Several models of translation, such as the ribosome flow model and other TASEP-based models, have been used to justify the role of codon ordering in determining spatial patterns of ribosomes along mRNAs (Reuveni et al., 2011; Tuller et al., 2011). Such models of translation consider each mRNA in isolation of all others, assuming a constant rate of initiation. In other words, TASEP models implicitly assume a constant, inexhaustible supply of free ribosomes and free tRNAs in the cell, so that the 5′ end of each mRNA is typically saturated with ribosomes (Reuveni et al., 2011), and the speed of elongation then sets the pace of protein production. Such models make sense only if ribosomes are in overabundance in the cell. As a result, the total number of ribosomes bound to mRNAs estimated by such models (>5×10^5, Extended Experimental Procedures) exceeds the empirical measurement of the total number of ribosomes in a yeast cell ($1.87 \times 10^5 \pm 5.6 \times 10^4$; von der Haar, 2008) by a factor of 2.5.

When we artificially increase the number of ribosomes and tRNAs in our simulations beyond their empirically measured abundances, we can recapitulate the patterns produced by TASEP models of translation (Figure S5A). In this regime, which we argue is unrealistic, we still observe a decrease in the average ribosome density with codon position, but this ramp is caused by collisions along each mRNA, and it persists regardless of gene-specific initiation probabilities or codon ordering within genes (Figure S5B). Thus, models of translation in both initiation- and elongation-limited regimes produce similar global patterns of ribosomal densities with codon position but for entirely different and contradictory mechanisms. Only the initiation-limited regime is consistent with empirical measurements of ribosome abundances in the yeast cell.

Ribosomal Interference and Codon Usage

Our simulations allow us to estimate the amount of time a ribosome spends waiting for a tRNA at each codon position, called ribosomal pausing, and also the amount of time a ribosome wastes at any position due to interference by an adjacent downstream ribosome that prevents further elongation, called ribosomal stalling. We identified the sequence features of a gene that predispose it to ribosomal pausing or stalling (Experimental Procedures).

Using GFP as an example transgene simulated at 50% mRNA transcriptome abundance, we found that increasing the transgene's codon bias tends to decrease the overall density of ribosomes on its mRNAs, as well as the frequency of ribosomal stalling (Figure 6). For a transgene with high CAI, the probability of finding a ribosome bound at a given codon is negatively correlated with the abundance of corresponding iso-accepting tRNAs (Pearson correlation, $R = -0.802$), but this correlation is much weaker for a transgene with low CAI ($R = 0.042$ and $p > 0.05$). In other words, the waiting time per codon is largely determined by the abundance of corresponding tRNAs for a gene with high CAI. But for a gene with low CAI, ribosomes densities are higher overall and so the waiting time at each codon is also influenced by interference with downstream ribosomes and, therefore, is not easily predicted from tRNA abundances. In fact, regardless of CAI, there is a strong correlation between ribosomal stalling at a position and the probability of ribosomal pausing 10 codons downstream ($R = 0.958$ for high CAI and $R = 0.644$ for low CAI). Because the probability of pausing in a high-CAI transgene sequence is correlated with tRNA abundances, it is possible to predict the positions of ribosomal stalling from the transgene

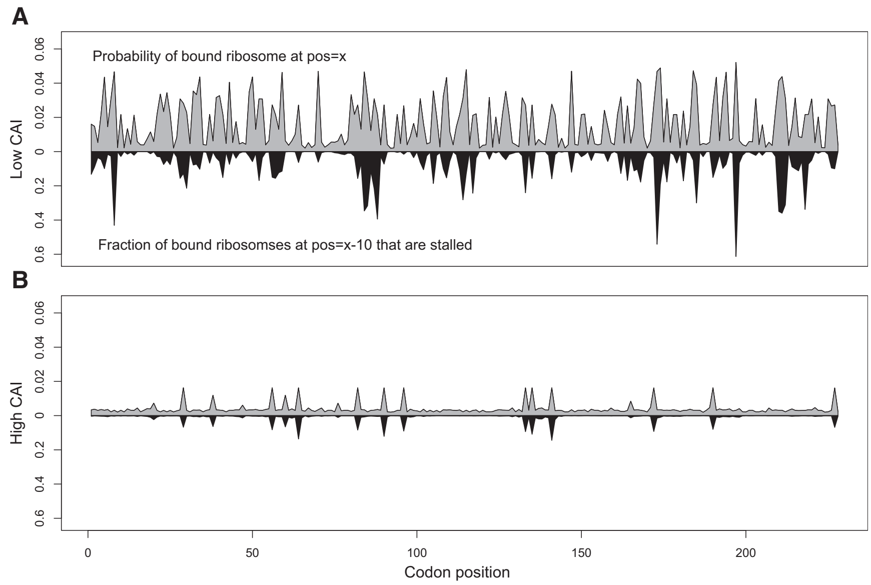

Figure 6. Codon Usage and Ribosomal Pausing and Stalling

(A and B) The influence of codon usage and amino acid sequence on ribosomal pausing and stalling for a simulated transgene with either (A) low or (B) high codon adaptation, expressed at 50% transcript abundance. Gray bars indicate the probability of finding a ribosome bound at a given codon position *x*, and black bars indicate the probability of finding a ribosome stalled at position x-10 (i.e., a ribosome whose further elongation is obstructed by another ribosome). High codon adaptation reduces both ribosome density and ribosome interference. The probability of a ribosome stalling at a position correlates strongly with the probability of a ribosome pausing 10 positions ahead ($R = 0.947$ for high CAI and $R = 0.644$ for low CAI). For a transgene with high CAI, the probability of finding a ribosome bound at a given codon position is strongly anticorrelated with the abundance of iso-accepting tRNAs for that codon ($R = 0.786$), but not for a transgene with low CAI ($R = -0.042$).

sequence alone. Understanding the effects of amino acid and codon usage on pausing and stalling may prove useful in designing transgene sequences to minimize ribosomal interference on its mRNAs.

Protein Translation under Stress

The simulations of translation described above were performed under parameters of optimal cell growth. Translation dynamics likely differ when a cell experiences stress. To investigate how protein production is affected by stress and how a cell might adapt in response, we simulated translation under conditions of amino acid starvation. We modeled starvation of a particular amino acid by reducing the abundance of its (charged) cognate tRNAs by either 2-, 5-, or 10-fold. As expected, we found that the rate of total protein production decreases under stress (Figures 7A and S6A). Furthermore, starvation of different amino acids can have radically different effects on protein production. For example, 10-fold starvation of amino acids Ala, Leu, Glu, Gln, or Ser decreases total protein production by at least 10-fold, whereas an equivalent starvation of Met, Trp, or His reduces protein production by less than 25% (Figure 7A). As expected, the effect of starvation of a particular amino acid is significantly correlated with its abundance encoded in the transcriptome ($p < 0.01$ in all cases).

Our simulations reveal that decreased protein synthesis upon starvation is caused primarily by a decrease in the pool of free ribosomes (Figures S6A and S6B). When tRNAs corresponding to a specific amino acid are in short supply, elongation of their codons becomes rate limiting, as has been predicted theoretically (Elf et al., 2003) and observed experimentally (Welch et al., 2009). As our simulations demonstrate, this effect creates traffic jams that increase the density of ribosomes on all mRNAs and increase the fraction of bound ribosomes that are stalled (Figure S6D). The increased density of bound ribosomes in turn decreases the pool of free tRNAs of all species, as each bound ribosome sequesters one tRNA in its P site. At equilibrium, the limited pool of free ribosomes and tRNAs reduces the initiation and elongation rates of all transcripts (Figure S6C) and hence retards total protein production.

Eukaryotic cells have evolved mechanisms to cope with stress, which we can analyze mechanistically using our model of translation. During amino acid starvation, eukaryotic cells respond (1) by repressing the production of ribosomal proteins and rRNAs (Moehle and Hinnebusch, 1991) and (2) by phosphorylating eIF2α by GCN2, which retards the formation of initiation complexes (Krishnamoorthy et al., 2001; Zhang et al., 2002; Hinnebusch and Lorsch, 2012). In order to study these adaptive responses, we simulated the repression of ribosomes by reducing the total number of ribosomes in the cell, and we simulated the phosphorylation of eIF2α by reducing the initiation probabilities of all genes by a fixed factor. Under mild stress conditions (2- to 5-fold decrease in charged tRNAs), reducing either the ribosome abundance or initiation probabilities was detrimental to protein production (Figure S7 and Table S5). However, when the cell experiences severe amino acid starvation, reducing ribosome abundance or initiation probabilities can partly rescue protein production (Figures 7B and S7 and Table S5). This increase in protein production, albeit not to the levels of the wild-type cell, is quite significant. This counterintuitive behavior can be explained by the fact that, under severe stress conditions, the cell becomes elongation limited instead of initiation limited. As a result, reducing the initiation rates of genes not only increases the pool of free ribosomes (Table S5) but also the pool of free tRNAs, especially the ones corresponding to the starved amino acid. This leads to an increase in the elongation rate of all genes and, hence, overall protein production.

Stress-induced repression of ribosomes and phosphorylation of eIF2α have previously been thought to be adaptive because they minimize resource waste. Our simulations indicate that such responses may also have a direct benefit of rescuing protein production and therefore increasing cell growth.

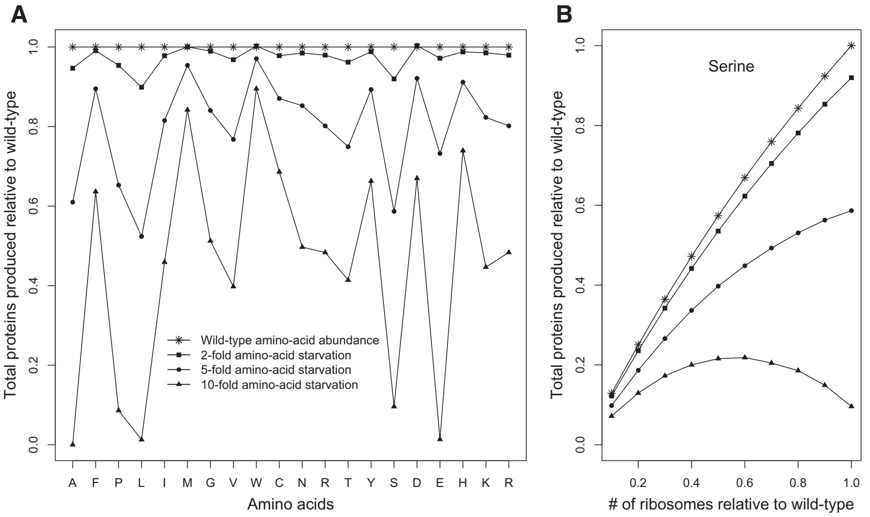

Figure 7. The Effects of Amino Acid Starvation and Cellular Stress Response on Protein Production

(A) For each amino acid, we reduced the relative abundance of its (charged) cognate tRNAs by either 2-, 5-, or 10-fold and then simulated translation in the cell. Although a stronger stress always leads to lower protein production, the strength of this effect varies widely among amino acids.

(B) During starvation, cells often respond by repressing ribosome production, which we modeled by reducing the total number of ribosomes in the cell and resimulating translation. Reducing ribosomes under normal or mild stress conditions always reduces protein production. However, during severe stress, reducing ribosomes by a moderate amount can partly rescue protein production, as in the case of serine shown in (B).

See also Figures S6 and S7 and Table S5.

DISCUSSION

We have used a whole-cell simulation model to study the dynamics of translation. This approach allows us to map the parameter regimes in which high codon adaptation is expected to increase transgene protein yield and by what mechanisms—revealing the critical role of free ribosomes in constraining initiation and protein production. This approach also elucidates the basic determinants of translation dynamics in the endogenous yeast transcriptome, providing estimates of initiation probabilities for all abundant yeast mRNAs. We have found a strong correlation between ORF length and initiation probability, which, we argue, provides a simple explanation for the apparent ramp of 5′ ribosome densities observed in ribosomal profiling data.

Whether endogenous protein production is initiation or elongation limited remains actively debated (Gingold and Pilpel, 2011; Plotkin and Kudla, 2011). It cannot easily be determined a priori which process should be limiting because the cellular abundances of some tRNA species are comparable to the abundance of ribosomes. Nonetheless, a long string of early experiments by Andersson and others established the empirical fact that initiation limits production for most endogenous proteins in healthy cells (Andersson and Kurland, 1990; Bulmer, 1991). Our simulations—and especially our results on how slow codons in an abundant mRNA retard protein production by depleting free ribosomes (Figure 2)—confirm and quantify the longstanding initiation-limited view of protein synthesis. Moreover, from an evolutionary perspective, it makes more sense for a cell to err on the side of producing a slight excess of tRNAs as opposed to an excess of ribosomes because ribosomes are much more costly to synthesize than tRNAs. Finally, it is important to note that the TASEP-based models of translation (e.g., Reuveni et al., 2011) cannot, even in principle, be used to assess whether protein production is limited by available ribosomes because such models assume a fixed, inexhaustible supply of free ribosomes. Nor can such models, which treat each mRNA molecule independently, assess how the codon usage of a transgene influences the pool of free ribosomes in a cell and thus feeds back to alter initiation rates of all transcripts and cell growth.

Although our simulations allow us to quantify translation dynamics in a cell, our model makes many simplifying assumptions, as mentioned previously. For instance, we assume that the total numbers of ribosomes, tRNAs, and mRNAs remain constant, which we have argued is a reasonable approximation based on empirical data (García-Martínez et al., 2004; Larson et al., 2011). Nonetheless, spatial heterogeneities in the distributions of tRNAs, mRNAs, and ribosomes (Reid and Nicchitta, 2012; Qian et al., 2012), which our model neglects, could modulate the effective diffusion constants of those molecules. We have also assumed that, upon elongation, a free tRNA is instantly recharged and available for further translation. Although this assumption is clearly violated in reality, tRNA charging is generally thought not to limit protein production, with about 80% of all tRNAs charged at all times due to strong negative feedback on aminoacyl synthetases (Varshney et al., 1991; Jakubowski and Goldman, 1992; Chu et al., 2011) (but see Brackley et al. [2011] and Qian et al. [2012]). Nonetheless, in conditions of amino acid starvation, the availability of charged tRNAs may become a limiting factor in protein production (Elf et al., 2003; Welch et al., 2009), as reflected by our simulations of translation under stress.

Our model also ignores the details of termination, as well as translation errors. Although missense errors do not affect overall protein yield or the pool of free ribosomes, such errors can reduce the amount of "functional" protein produced or even produce detrimental, misfolded protein products (Drummond and Wilke, 2008, 2009). Systematically predicting which mutations will cause nonfunctional or deleterious protein folds is not feasible, but nonetheless, mistranslation remains a strong force of selection on codon usage over evolutionary timescales (Drummond and Wilke, 2008, 2009). By contrast, premature termination or nonsense errors affect both protein yield and the pool of free ribosomes. Because the probability of a nonsense error at a codon is inversely proportional to the amount of tRNAs available (Gilchrist, 2007; Shah and Gilchrist, 2010), incorporating

nonsense errors into our model would tend to exaggerate the effects of CAI and mRNA abundance on protein yield.

Aside from the systematic processes described above, our model also neglects a host of other sequence-specific features that are known to influence protein production and cellular fitness in specific cases, such as cotranslational requirements for ribosomal pausing (Kimchi-Sarfaty et al., 2007), internal mRNA structures that may retard elongation (Tuller et al., 2010b), synonymous codons required for proper splicing (Chamary et al., 2006), the effects of tRNA isoforms, neighboring codon interactions, and the recently discovered rRNA-mRNA interactions that operate in *E. coli*, but not in yeast (Li et al., 2012). Although each of these effects has been observed in a few empirical cases, it is difficult to predict when they will operate and what consequences they will have in general. Like all models, our model of translation should be particularly useful when it fails to match measurements of protein production for individual transcripts, indicating the action of some factor missing from the model that influences the translation of a particular gene. Nonetheless, these types of highly sequence-specific factors are unlikely to alter the general conclusions we have drawn from our model, such as the predominant role of free ribosomes in setting the overall pace of translation and the role of initiation rates in causing a ramp of 5′ ribosome densities.

EXPERIMENTAL PROCEDURES

S. cerevisiae Transcriptome

To define the mRNA transcriptome, we selected the 3,795 genes from *S. cerevisiae* (S288c June 6, 2008 release; Cherry et al., 2012) for which Ingolia et al. (2009) obtained reliable estimates of average ribosomal densities. We fixed the total number of mRNAs to 60,000 (Zenklusen et al., 2008) and sampled mRNAs based on the relative abundances measured by Ingolia et al. (2009), ensuring that each gene had at least one mRNA represented in the transcriptome. mRNA abundances ranged from 1 to 1,254 molecules per gene (Tables 1 and S1). The (mRNA) transcriptome size was then defined as the total number of nucleotides comprised by the 60,000 mRNA molecules.

Generating Transgenes with Various CAI Values

We generated nucleotide sequences of GFP and other transgenes with various different CAI values. To produce a specified CAI value, we calculated relative synonymous codon usage (RSCU) in *S. cerevisiae* from 134 ribosomal genes (Table S3) (Sharp and Li, 1987). We then sampled codons based on RSCU. There are typically many nucleotide sequences with the same, or very similar, CAI values. Thus, for each simulation involving transgenes, we used ten sequences of similar CAI values and equal mRNA abundances to represent the transgene, in order to alleviate noisy, sequence-specific effects.

Calculating 5′ Folding Energy

Coding sequences and UTRs for *S. cerevisiae* were downloaded from Ensemble (http://www.ensemblgenomes.org). We removed sequences with lengths not equal to a multiple of three, with premature stop codons, or with a continuous string of >3 ambiguous N symbols. We used RNAfold (Hofacker et al., 1994) to estimate the mRNA folding energy from base −4 to 37 for each gene, using default parameters.

Estimating Ribosomal Interference

To identify regions of ribosomal pausing and interference on a transgene sequence, we simulated translation in the cell with a transgene accounting for 50% of the (mRNA) transcriptome. We ran the simulation for 500 s in equilibrium and sampled the state of the system every second. We used the average number of ribosomes bound at each position to quantify the frequency of ribosomal pausing. To quantify the frequency of ribosomal stalling, we calculated the fraction of bound ribosomes at a position that also have another bound ribosome ten codons (positions) ahead on that mRNA in the same time sample.

SUPPLEMENTAL INFORMATION

Supplemental Information includes Extended Experimental Procedures, seven figures, two data files, and five tables and can be found with this article online at http://dx.doi.org/10.1016/j.cell.2013.05.049.

LICENSING INFORMATION

This is an open-access article distributed under the terms of the Creative Commons Attribution License, which permits unrestricted use, distribution, and reproduction in any medium, provided the original author and source are credited.

ACKNOWLEDGMENTS

We thank M.A. Gilchrist, S.S. Yadavalli, and S. Jagannathan. J.B.P. acknowledges support from the Burroughs Wellcome Fund, the David and Lucile Packard Foundation, the James S. McDonnell Foundation, the Alfred P. Sloan Foundation, the Foundational Questions in Evolutionary Biology Fund (RFP-12-16), and grant D12AP00025 from the U.S. Department of the Interior. M.N. and G.K. were supported by The Wellcome Trust grant 077248 to David Tollervey and also thank the Tollervey lab. M.N. was supported by the Darwin Trust of Edinburgh.

Received: June 16, 2012
Revised: February 11, 2013
Accepted: May 29, 2013
Published: June 20, 2013

REFERENCES

Andersson, S.G., and Kurland, C.G. (1990). Codon preferences in free-living microorganisms. Microbiol. Rev. *54*, 198–210.

Arava, Y., Wang, Y., Storey, J.D., Liu, C.L., Brown, P.O., and Herschlag, D. (2003). Genome-wide analysis of mRNA translation profiles in Saccharomyces cerevisiae. Proc. Natl. Acad. Sci. USA *100*, 3889–3894.

Brackley, C.A., Romano, M.C., and Thiel, M. (2011). The dynamics of supply and demand in mRNA translation. PLoS Comput. Biol. *7*, e1002203.

Bulmer, M. (1991). The selection-mutation-drift theory of synonymous codon usage. Genetics *129*, 897–907.

Cannarozzi, G., Schraudolph, N.N., Faty, M., von Rohr, P., Friberg, M.T., Roth, A.C., Gonnet, P., Gonnet, G., and Barral, Y. (2010). A role for codon order in translation dynamics. Cell *141*, 355–367.

Chamary, J.V., Parmley, J.L., and Hurst, L.D. (2006). Hearing silence: non-neutral evolution at synonymous sites in mammals. Nat. Rev. Genet. *7*, 98–108.

Chan, P.P., and Lowe, T.M. (2009). GtRNAdb: a database of transfer RNA genes detected in genomic sequence. Nucleic Acids Res. *37*(Database issue), D93–D97.

Cherry, J.M., Hong, E.L., Amundsen, C., Balakrishnan, R., Binkley, G., Chan, E.T., Christie, K.R., Costanzo, M.C., Dwight, S.S., Engel, S.R., et al. (2012). Saccharomyces Genome Database: the genomics resource of budding yeast. Nucleic Acids Res. *40*(Database issue), D700–D705.

Chu, D., and von der Haar, T. (2012). The architecture of eukaryotic translation. Nucleic Acids Res. *40*, 10098–10106.

Chu, D., Barnes, D.J., and von der Haar, T. (2011). The role of tRNA and ribosome competition in coupling the expression of different mRNAs in Saccharomyces cerevisiae. Nucleic Acids Res. *39*, 6705–6714.

de Smit, M.H., and van Duin, J. (1990). Secondary structure of the ribosome binding site determines translational efficiency: a quantitative analysis. Proc. Natl. Acad. Sci. USA *87*, 7668–7672.

Ding, Y., Shah, P., and Plotkin, J.B. (2012). Weak 5′-mRNA secondary structures in short eukaryotic genes. Genome Biol. Evol. *4*, 1046–1053.

Drummond, D.A., and Wilke, C.O. (2008). Mistranslation-induced protein misfolding as a dominant constraint on coding-sequence evolution. Cell *134*, 341–352.

Drummond, D.A., and Wilke, C.O. (2009). The evolutionary consequences of erroneous protein synthesis. Nat. Rev. Genet. *10*, 715–724.

Duan, J., Wainwright, M.S., Comeron, J.M., Saitou, N., Sanders, A.R., Gelernter, J., and Gejman, P.V. (2003). Synonymous mutations in the human dopamine receptor D2 (DRD2) affect mRNA stability and synthesis of the receptor. Hum. Mol. Genet. *12*, 205–216.

Elf, J., Nilsson, D., Tenson, T., and Ehrenberg, M. (2003). Selective charging of tRNA isoacceptors explains patterns of codon usage. Science *300*, 1718–1722.

Eyre-Walker, A., and Bulmer, M. (1993). Reduced synonymous substitution rate at the start of enterobacterial genes. Nucleic Acids Res. *21*, 4599–4603.

Gallie, D.R. (1998). A tale of two termini: a functional interaction between the termini of an mRNA is a prerequisite for efficient translation initiation. Gene *216*, 1–11.

García-Martínez, J., Aranda, A., and Pérez-Ortín, J.E. (2004). Genomic run-on evaluates transcription rates for all yeast genes and identifies gene regulatory mechanisms. Mol. Cell *15*, 303–313.

Gilchrist, M.A. (2007). Combining models of protein translation and population genetics to predict protein production rates from codon usage patterns. Mol. Biol. Evol. *24*, 2362–2372.

Gilchrist, M.A., and Wagner, A. (2006). A model of protein translation including codon bias, nonsense errors, and ribosome recycling. J. Theor. Biol. *239*, 417–434.

Gingold, H., and Pilpel, Y. (2011). Determinants of translation efficiency and accuracy. Mol. Syst. Biol. *7*, 481.

Gustafsson, C., Govindarajan, S., and Minshull, J. (2004). Codon bias and heterologous protein expression. Trends Biotechnol. *22*, 346–353.

Hall, M.N., Gabay, J., Débarbouillé, M., and Schwartz, M. (1982). A role for mRNA secondary structure in the control of translation initiation. Nature *295*, 616–618.

Hinnebusch, A.G., and Lorsch, J.R. (2012). The mechanism of eukaryotic translation initiation: new insights and challenges. Cold Spring Harb. Perspect. Biol. *4*.

Hofacker, I.L., Fontana, W., and Stadler, P. (1994). Fast folding and comparison of RNA secondary structures. Monatsh. Chem. *125*, 167–188.

Hurowitz, E.H., and Brown, P.O. (2003). Genome-wide analysis of mRNA lengths in Saccharomyces cerevisiae. Genome Biol. *5*, R2.

Ingolia, N.T., Ghaemmaghami, S., Newman, J.R.S., and Weissman, J.S. (2009). Genome-wide analysis in vivo of translation with nucleotide resolution using ribosome profiling. Science *324*, 218–223.

Jackson, R.J., Hellen, C.U.T., and Pestova, T.V. (2010). The mechanism of eukaryotic translation initiation and principles of its regulation. Nat. Rev. Mol. Cell Biol. *11*, 113–127.

Jakubowski, H., and Goldman, E. (1992). Editing of errors in selection of amino acids for protein synthesis. Microbiol. Rev. *56*, 412–429.

Kimchi-Sarfaty, C., Oh, J.M., Kim, I.-W., Sauna, Z.E., Calcagno, A.M., Ambudkar, S.V., and Gottesman, M.M. (2007). A "silent" polymorphism in the MDR1 gene changes substrate specificity. Science *315*, 525–528.

Krishnamoorthy, T., Pavitt, G.D., Zhang, F., Dever, T.E., and Hinnebusch, A.G. (2001). Tight binding of the phosphorylated α subunit of initiation factor 2 (eIF2α) to the regulatory subunits of guanine nucleotide exchange factor eIF2B is required for inhibition of translation initiation. Mol. Cell. Biol. *21*, 5018–5030.

Kudla, G., Murray, A.W., Tollervey, D., and Plotkin, J.B. (2009). Coding-sequence determinants of gene expression in Escherichia coli. Science *324*, 255–258.

Lackner, D.H., Beilharz, T.H., Marguerat, S., Mata, J., Watt, S., Schubert, F., Preiss, T., and Bähler, J. (2007). A network of multiple regulatory layers shapes gene expression in fission yeast. Mol. Cell *26*, 145–155.

Larson, D.R., Zenklusen, D., Wu, B., Chao, J.A., and Singer, R.H. (2011). Real-time observation of transcription initiation and elongation on an endogenous yeast gene. Science *332*, 475–478.

Li, G.-W., Oh, E., and Weissman, J.S. (2012). The anti-Shine-Dalgarno sequence drives translational pausing and codon choice in bacteria. Nature *484*, 538–541.

Lin, Z., and Li, W.-H. (2012). Evolution of 5′ untranslated region length and gene expression reprogramming in yeasts. Mol. Biol. Evol. *29*, 81–89.

Mitarai, N., Sneppen, K., and Pedersen, S. (2008). Ribosome collisions and translation efficiency: optimization by codon usage and mRNA destabilization. J. Mol. Biol. *382*, 236–245.

Moehle, C.M., and Hinnebusch, A.G. (1991). Association of RAP1 binding sites with stringent control of ribosomal protein gene transcription in Saccharomyces cerevisiae. Mol. Cell. Biol. *11*, 2723–2735.

Plotkin, J.B., and Kudla, G. (2011). Synonymous but not the same: the causes and consequences of codon bias. Nat. Rev. Genet. *12*, 32–42.

Politz, J.C.R., Tuft, R.A., and Pederson, T. (2003). Diffusion-based transport of nascent ribosomes in the nucleus. Mol. Biol. Cell *14*, 4805–4812.

Qian, W., Yang, J.-R., Pearson, N.M., Maclean, C., and Zhang, J. (2012). Balanced codon usage optimizes eukaryotic translational efficiency. PLoS Genet. *8*, e1002603.

Reid, D.W., and Nicchitta, C.V. (2012). Primary role for endoplasmic reticulum-bound ribosomes in cellular translation identified by ribosome profiling. J. Biol. Chem. *287*, 5518–5527.

Reuveni, S., Meilijson, I., Kupiec, M., Ruppin, E., and Tuller, T. (2011). Genome-scale analysis of translation elongation with a ribosome flow model. PLoS Comput. Biol. *7*, e1002127.

Salis, H.M., Mirsky, E.A., and Voigt, C.A. (2009). Automated design of synthetic ribosome binding sites to control protein expression. Nat. Biotechnol. *27*, 946–950.

Shah, P., and Gilchrist, M.A. (2010). Effect of correlated tRNA abundances on translation errors and evolution of codon usage bias. PLoS Genet. *6*.

Shah, P., and Gilchrist, M.A. (2011). Explaining complex codon usage patterns with selection for translational efficiency, mutation bias, and genetic drift. Proc. Natl. Acad. Sci. USA *108*, 10231–10236.

Sharp, P.M., and Li, W.-H. (1987). The codon Adaptation Index—a measure of directional synonymous codon usage bias, and its potential applications. Nucleic Acids Res. *15*, 1281–1295.

Siwiak, M., and Zielenkiewicz, P. (2010). A comprehensive, quantitative, and genome-wide model of translation. PLoS Comput. Biol. *6*, e1000865.

Tanguay, R.L., and Gallie, D.R. (1996). Translational efficiency is regulated by the length of the 3′ untranslated region. Mol. Cell. Biol. *16*, 146–156.

Tuller, T., Carmi, A., Vestsigian, K., Navon, S., Dorfan, Y., Zaborske, J., Pan, T., Dahan, O., Furman, I., and Pilpel, Y. (2010a). An evolutionarily conserved mechanism for controlling the efficiency of protein translation. Cell *141*, 344–354.

Tuller, T., Waldman, Y.Y., Kupiec, M., and Ruppin, E. (2010b). Translation efficiency is determined by both codon bias and folding energy. Proc. Natl. Acad. Sci. USA *107*, 3645–3650.

Tuller, T., Veksler-Lublinsky, I., Gazit, N., Kupiec, M., Ruppin, E., and Ziv-Ukelson, M. (2011). Composite effects of gene determinants on the translation speed and density of ribosomes. Genome Biol. *12*, R110.

Varshney, U., Lee, C.P., and RajBhandary, U.L. (1991). Direct analysis of aminoacylation levels of tRNAs in vivo. Application to studying recognition of Escherichia coli initiator tRNA mutants by glutaminyl-tRNA synthetase. J. Biol. Chem. *266*, 24712–24718.

von der Haar, T. (2008). A quantitative estimation of the global translational activity in logarithmically growing yeast cells. BMC Syst. Biol. *2*, 87.

Waldron, C., and Lacroute, F. (1975). Effect of growth rate on the amounts of ribosomal and transfer ribonucleic acids in yeast. J. Bacteriol. *122*, 855–865.

Warner, J.R. (1999). The economics of ribosome biosynthesis in yeast. Trends Biochem. Sci. *24*, 437–440.

Welch, M., Govindarajan, S., Ness, J.E., Villalobos, A., Gurney, A., Minshull, J., and Gustafsson, C. (2009). Design parameters to control synthetic gene expression in Escherichia coli. PLoS ONE *4*, e7002.

Werner, A. (2011). Predicting translational diffusion of evolutionary conserved RNA structures by the nucleotide number. Nucleic Acids Res. *39*, e17.

Zenklusen, D., Larson, D.R., and Singer, R.H. (2008). Single-RNA counting reveals alternative modes of gene expression in yeast. Nat. Struct. Mol. Biol. *15*, 1263–1271.

Zhang, P., McGrath, B.C., Reinert, J., Olsen, D.S., Lei, L., Gill, S., Wek, S.A., Vattem, K.M., Wek, R.C., Kimball, S.R., et al. (2002). The GCN2 eIF2α kinase is required for adaptation to amino acid deprivation in mice. Mol. Cell. Biol. *22*, 6681–6688.

EXTENDED EXPERIMENTAL PROCEDURES

Simulation Model

We describe protein translation using a discrete-state continuous-time Markov model of initiation, elongation, and termination events in a cell. The model assumes a fixed total number of ribosomes and tRNAs, and it describes how these entities initiate and elongate a fixed supply of mRNAs. Our model neglects the dynamics of transcription, mRNA decay, and co-transcriptional translation; it also neglects the production and decay of ribosomes and tRNAs themselves. These processes are typically slower than the dynamics of translation, and so our model nonetheless provides an accurate description of translation in a cell in most conditions.

We assume a genome comprised of n genes, each with a prescribed coding sequence, and each with a fixed abundance A_i, of mRNA copies in the cell. Gene i encodes an mRNA of length L_i codons; each such codon is assigned one of k possible values (k = 61 in the standard genetic code). Each gene i also has a corresponding probability of translation initiation, denoted p_i, which is described below.

Corresponding to each type of codon j is one of 41 iso-accepting tRNA species, denoted $\phi(j)$, which has a fixed total abundance $T^t_{\phi(j)}$ in the cell. At any time in our Markov model, each molecule of tRNA species $\phi(j)$ is either free in the cell, or bound, along with a ribosome, to some codon of type j in some mRNA in the cell. Thus, at each time, the total number of tRNAs of type $\phi(j)$ can be decomposed into those that are currently bound and those that are currently free: $T^t_{\phi(j)} = T^b_{\phi(j)} + T^f_{\phi(j)}$. Likewise, the total number of ribosomes, R^t, can be decomposed into bound and free: $R^t = R^b + R^f$. Moreover, the number of bound ribosomes always equals the total number of bound tRNAs of all species: $R^b = \sum_{k=1}^{41} T^b_k$.

In our continuous-time Markov model, initiation and elongation events occur at rates that are determined by the current state of system (the number of free ribosomes, and the locations of all bound ribosomes) and by the underlying physical parameters of the cell. The underlying physical parameters are simply the volume of the cell, and the characteristic lengths and diffusion constants of ribosomes and tRNA molecules. The time between subsequent events are exponentially distributed, and Monte Carlo simulations proceed simply by incrementing time according to exponential deviates and re-computing rates of subsequent events (Gillespie, 1977). We provide the model source code, and associated datasets used in the current simulations as a supplement (Data S1). Additionally, the latest version of the code is also made freely available at http://mathbio.sas.upenn.edu/shah-cell-2013-code.tar.gz.

Diffusion of Ribosomes and tRNAs

We compute initiation and elongation rates by considering the diffusion of ribosome and tRNA molecules in the cell. Assuming a spherical cell of volume $V = 4.2\times10^{-17}$ m^3 (Jorgensen et al., 2002), the number of different discrete positions that can be occupied by any molecule is $N = V/\lambda^3$, where λ is the characteristic length of the molecule. The characteristic lengths of tRNA and ribosomes have been measured as $\lambda_t = 1.5\times10^{-8}$ m and $\lambda_r = 3\times10^{-8}$ m, respectively (Nissen et al., 1999; Politz et al., 2003). Thus, the number of available discrete positions for tRNA and ribosome molecules are $N_t = 1.24\times10^7$ and $N_r = 1.56\times10^6$, respectively.

The average time required for any given molecule to move from one position in the cell to another, known as the characteristic time τ, is given by

$$\tau = \frac{\lambda^2}{6D} \tag{1}$$

where D is the diffusion coefficient of the molecule. The diffusion coefficients of tRNAs and ribosomes are known, $D_t = 8.42\times10^{-11}$ m^2/s and $D_r = 3\times10^{-13}$ m^2/s (Politz et al., 2003; Werner, 2011), and hence their characteristic times are $\tau_t = 4.45\times10^{-7}$ s and $\tau_r = 5\times10^{-4}$ s, respectively. The characteristic times allow us to compute the rate at which a free ribosome or mRNA molecule reaches any particular position in the cell. In particular, if there are N positions that can be occupied by a molecule, then a given molecule with characteristic time τ will reach a particular position in the cell at rate $1/\tau N$. For example, if there are R^f free ribosomes, then the rate at which any free ribosome reaches a given mRNA molecule is simply $R^f/\tau N_r$.

Translation Initiation Rates

Given the current state of the system (the number of free ribosomes, and the locations of all bound ribosomes), each mRNA of type i will be initiated at rate ρ_i. The rate ρ_i is set to zero if any of the first 10 codons of the mRNA is currently bound by a ribosome. Otherwise, the rate is

$$\rho_i = p_i \frac{R^f}{\tau N_r}.$$

The term $R^f/\tau N_r$ in this equation denotes the rate at which any free ribosome diffuses to a given mRNA molecule. And the term p_i denotes the initial probability of an mRNA of type i: the chance that a ribosome will actually initiate translation of such an mRNA molecule, once it has diffused to its 5′ end. The parameters p_i allow us to account for sequence-specific variation in initiation probabilities among genes (Kudla et al., 2009).

Translation Elongation Rates

Any given ribosome currently bound to some mRNA will elongate at some rate. Consider a ribosome bound at codon position k on an mRNA. Its rate of elongation is set to zero if any of the following k + 10 codons of the mRNA are currently occupied by another ribosome, because of interference. Otherwise, the rate at which the ribosome elongates the subsequent codon, of type j, depends on the

number of free cognate tRNAs for that codon $T^f_{\phi(j)}$ and the wobble parameter associated with the tRNA-codon pair w_j. If there is a perfect match between the tRNA and the codon, then $w_j = 1$. Else $w_{ry/yr} = 0.64$ if the mismatch is due to a purine-pyrimidine wobble or $w_{rr/yy} = 0.61$ if the mismatch is due to purine-purine or pyrimidine-pyrimidine wobble (Curran and Yarus, 1989; Lim and Curran, 2001). The rate at which a cognate tRNA elongates to the codon at position $k+1$ is thus given by

$$\frac{T^f_{\phi(j)} w_j}{\tau_t N_t}$$

In addition, during elongation various tRNAs compete for the focal ribosome. The ribosome thus spends a considerable amount of time checking whether a given tRNA in its *A*-site is in fact a cognate tRNA for the codon it is about to elongate. The time spent by the ribosome in selecting the cognate tRNA depends on the relative abundances of various tRNAs as well as organism specific kinetic rates associated with ribosomal proofreading. Because these kinetic rates are not available for yeast, we use the values obtained in *Escherichia coli* (Fluitt et al., 2007; Gromadski and Rodnina, 2004). Using these parameters and tRNA abundances in yeast, we used numerical simulations described in Fluitt et al. (2007) to estimate the average time spent by the ribosome in kinetic proofreading to select the correct tRNA. As a result, accounting for tRNA competition coefficient *s*, the actual elongation rate of a codon is

$$\frac{T^f_{\phi(j)} w_j s}{\tau_t N_t}$$

Translation Termination

We assume that translation termination is an instantaneous event that occurs immediately after elongation of the last codon at position *L*. Upon termination the pool of free ribosomes and free tRNAs corresponding to the codon *j′* at position $L-1$ each increases by 1 $(R^f \rightarrow R^f + 1;\ T^f_{\phi(j)'} \rightarrow T^f_{\phi(j)'} + 1)$.

Analytic Approximation for Steady-State Behavior

Whereas we have used the complete stochastic model described above to produce all the simulation figures in the main text, it is convenient to approximate its steady-state behavior by analytical equations, especially for the purpose of inferring gene-specific initiation probabilities from ribosomal profiling data. To do so we derive here an analytic steady-state approximation, based on ordinary differential equations that treat all quantities as continuous variables and are therefore accurate when the molecular quantities are large. This approximation neglects the possibility of ribosomal interference during elongation, and so it is not expected to hold in regimes for which mRNAs are densely packed with ribosomes. We will derive analytic approximations for the steady state elongation times of codons, the amount of free tRNAs, the initiation and total elongation times of all mRNAs, and the steady-state number of free ribosomes in the cell.

Consider a cell with a total number of ribosomes R^t and *n* genes each with A_i mRNA copies. Assuming no ribosomal interference during translation, the expected number of ribosomes bound to each mRNA can be approximated by solving the differential equation

$$\frac{dR^b_i}{dt} = \rho_i - R^b_i \epsilon_i \quad (2)$$

where ρ_i and ϵ_i are the rates of initiation and total elongation of the ith mRNA, respectively. At steady-state the total number of bound ribosomes is then given by

$$R^b = \sum_{i=1}^{n} \frac{A_i \rho_i}{\epsilon_i} \quad (3)$$

The rates of translation initiation and total elongation in turn depend on the amounts of free ribosomes R^f and free tRNAs T^f, in addition to the characteristic times of these molecules. We assume that translation termination is instantaneous and does not contribute to the overall rate of translation. Thus the initiation rate on an mRNA can be given as

$$\rho_i = \frac{R^f p_i}{\tau_r N_r} \quad (4)$$

where p_i is the probability of initiation given that the ribosome has reached the mRNA. p_i is sequence-specific and accounts for the variation in initiation rates of various mRNAs.

Similarly, when a ribosome is bound to the mRNA, the time taken to elongate codon *j* depends on the number of the free cognate tRNAs $T^f_{\phi(j)}$, the wobble parameter w_j, and the tRNA competition coefficient *s*:

$$c_j = \frac{\tau_t N_t}{T^f_{\phi(j)} w_j s}. \quad (5)$$

Thus at equilibrium, assuming no ribosomal collisions/interference, the expected total elongation rate of a ribosome on an mRNA is

$$\epsilon_i = \frac{1}{\sum_{j=1}^{k} x_j c_j} \tag{6}$$

where x_j is the number of codons of type j, and k denotes the total types of codons (typically $k = 61$).

Case 1: One Gene and One Amino Acid with Two Codons

Consider a simple case of one gene of length L codons composed of a single amino acid with two types of codons, each translated by a single tRNA type (T_1 or T_2). Let the expression level of the gene be A, relative frequency of codon 1 be u, and the total number of ribosomes in the cell R^t. Based on Equation (6), the total elongation rate of that gene is given by

$$\epsilon = \frac{1}{L(uc_1 + (1-u)c_2)} \tag{7}$$

where c_1 and c_2 are given by Equation (5)

$$c_1 = \frac{\tau_t N_t}{T_1^f w_1 s} \tag{8}$$

$$c_2 = \frac{\tau_t N_t}{T_2^f w_2 s} \tag{9}$$

Note that whenever a ribosome is bound to an mRNA waiting for a tRNA corresponding to the codon at its A-site, a tRNA is bound at its P-site attached to the growing polypeptide chain. Assuming that the codons in the gene are randomly distributed, the frequency of tRNA types at ribosomal P-sites are independent of the waiting time for codons in the A-sites. In addition, the total number of bound ribosomes should equal the number of bound tRNAs of all types $R^b = T_1^b + T_2^b$. As a result, the number of bound tRNAs of each type is simply proportional to its codon usage.

$$T_1^b = R^b u \tag{10}$$

$$T_2^b = R^b (1-u) \tag{11}$$

Note that the above relationship works if the number of bound ribosomes R^b is less than the ratio of total tRNAs of either type to their codon usage: $(R^b < \min(T_1^t/u, T_2^t/(1-u)))$. Therefore, by plugging Equations (8–11) in Equation (7) we get

$$\epsilon = \frac{1}{L\tau_t N_t \left(\frac{u}{T_1^f w_1 s} + \frac{1-u}{T_2^f w_2 s} \right)} \tag{12}$$

$$= \frac{1}{L\tau_t N_t \left(\frac{u}{w_1 s \left(T_1^t - R^b u\right)} + \frac{1-u}{w_2 s \left(T_2^t - R^b (1-u)\right)} \right)} \tag{13}$$

From the above Equations (8–13) it should be clear that in order to estimate the elongation times of codons, amount of free tRNAs, initiation and translation rate, it is sufficient to estimate the number of free ribosomes R^f. Given the fundamental parameters of the cell such as the cell volume, characteristic times of tRNAs and ribosomes, the total number of tRNAs and ribosomes, number of genes, their composition and their mRNA expression we can calculate the number of free ribosomes R^f by plugging Equations (4 and 13) in Equation (3).

$$R^b = \frac{A\rho}{\epsilon} \tag{14}$$

$$= \frac{R^f A p L \tau_t N_t}{\tau_r N_r s} \left(\frac{u}{w_1 \left(T_1^t - R^b u\right)} + \frac{1-u}{w_2 \left(T_2^t - R^b (1-u)\right)} \right) \tag{15}$$

$$R^t - R^f = \frac{R^f ApL\tau_t N_t}{\tau_r N_r}\left(\frac{u}{w_1\left(T_1^t-(R^t-R^f)u\right)}+\frac{1-u}{w_2\left(T_2^t-(R^t-R^f)(1-u)\right)}\right) \tag{16}$$

Upon simplification we get

$$R^f = \frac{R^t}{1+z\left(\frac{u}{w_1\left(T_1^t-\left(R^t-R^f\right)u\right)}+\frac{1-u}{w_2\left(T_2^t-\left(R^t-R^f\right)(1-u)\right)}\right)} \tag{17}$$

where

$$z = \frac{ApL\tau_t N_t}{\tau_r N_r s} \tag{18}$$

Thus, by solving the nonlinear Equation (17) we can compute the number of free ribosomes at equilibrium.

Case 2: Multiple Genes and Multiple Amino Acids with Varying Numbers of Codons

The above described Equation (17) can be easily expanded to its most general form of say 61 total codon types and n genes of length L_i each with A_i mRNAs, p_i initiation probabilities, and codon frequencies $\overrightarrow{u_i}$ as follows

$$R^f = \frac{R^t}{1+z\left(\sum_{j=1}^{61}\frac{u'_j}{w_j\left(T^t_{\phi(j)}-\left(R^t-R^f\right)\sum_{k|\phi(k)=\phi(j)}u'_k\right)}\right)} \tag{19}$$

where

$$z = \frac{\tau_t N_t}{\tau_r N_r s}\left(\sum_{i=1}^{n}A_i p_i L_i\right) \tag{20}$$

$$u'_j = \frac{\sum_{i=1}^{n}u_{j,i}A_i p_i L_i}{\sum_{i=1}^{n}A_i p_i L_i} \tag{21}$$

$$\sum_{j=1}^{61}u_{j,i} = 1 \tag{22}$$

$$\sum_{j=1}^{61}u'_j = 1 \tag{23}$$

Estimation of Gene-Specific Initiation Probabilities p_i

We can use our analytic approximations for the steady-state behavior in the cell to estimate gene-specific initiation probabilities from the ribosomal profiling data of Ingolia et al. (2009). The ribosome profiling data provide gene-specific equilibrium ribosomal densities R_i^b/A_i.

$$R_i^b = \frac{A_i \rho_i}{\epsilon_i} \tag{24}$$

$$= \frac{A_i R^f p_i \tau_t N_t L_i \sum_{j=1}^{61}\frac{u_{j,i}}{w_j\left(T^t_{\phi(j)}-T^b_{\phi(j)}\right)}}{\tau_r N_r s} \tag{25}$$

Therefore,

$$p_i = \frac{R_i^b \tau_r N_r s}{\left(R^t - \sum_i^n R_i^b\right) A_i L_i \tau_t N_t \left(\sum_{j=1}^{61} \frac{u_{j,i}}{w_j \left(T_{\phi(j)}^t - T_{\phi(j)}^b\right)}\right)} \tag{26}$$

Assuming about 85% of all ribosomes are bound to mRNAs (Arava et al., 2003) and that $T_{\phi(j)}^b \ll T_{\phi(j)}^t$ (Jakubowski and Goldman, 1984; Varshney et al., 1991; Chu et al., 2011), we can approximate the above equation as follows to infer the initiation probabilities p_i:

$$p_i \approx \frac{R_i^b x}{A_i L_i \left(\sum_{j=1}^{61} \frac{u_{j,i}}{w_j T_{\phi(j)}^t}\right)} \tag{27}$$

where

$$x = \frac{\tau_r N_r s}{0.15 R^t \tau_t N_t}$$

Effect of Transgene Abundance on Transcriptome Size

We explored transgene translation in two cases: one in which the transgene mRNA replaces cellular mRNAs keeping the total mRNA transcriptome size in nucleotides constant, and the other in which transgene mRNA is simply added to the endogenous mRNA, increasing the total mRNA transcriptome size. For scale, it is helpful to note that 1% of the mRNA in the transcriptome corresponds to 840 mRNAs of GFP.

Effect of Gene Length on Inferences of Initiation Probabilities

To investigate whether the negative correlation between gene length and inferred initiation probability observed in the yeast data is the result of any estimation bias we simulated a cell with 100 pairs of genes. Genes within each pair had the same codon composition, same CAI, and same initiation probabilities, but one gene was double the length of the other (the coding sequence was simply repeated). We simulated the translation of all pairs of genes, each assigned 300 mRNA copies in the cell (60,000 total mRNAs). Using the equilibrium ribosome densities of each simulated gene we then re-estimated their initiation probabilities. We found that doubling the gene length did not affect each gene's inferred initiation probability, as desired (Kolmogorov-Smirnov test, $p > 0.9$, Spearman correlation, $R = 0.997$).

In addition, we validated that we can reliably infer initiation probabilities from simulated ribosomal profiling data even when gene length and initiation probabilities are positively correlated (Figures S1C and S1D). This result indicates that the negative correlation between gene length and inferred initiation probability observed in the real yeast data is not an artifact of our inference procedure.

Correlation between Gene Length and Ribosome Density in Ribosome Profiling Data

One of the hallmark features of ribosomal profiling data (Ingolia et al., 2009) is the decrease in ribosome density with increasing codon position. This has been argued to be driven by heterogeneity in ribosome density along each mRNA molecule, with higher densities in the 5′ region of genes due to less optimal codons (Tuller et al., 2010). In order to show that position-specific heterogeneity in ribosome density is not in fact the primary cause of these patterns we used the average ribosomal density of each gene and assumed that this density is spread uniformly across the entire length of the sequence. We then recomputed the transcriptome-wide average ribosome density, by codon position, assuming a uniform density along each mRNA. We found that in the resulting profile, even upon removing position specific heterogeneity for each individual mRNA, we still observed a sharp decrease in average ribosome density with codon position (Figure S3A). In addition, when inspecting the profiling data on a gene-by-gene basis we find that just as many genes exhibit a trend of increasing ribosome density, from 5′ to 3′, as show evidence of decreasing ribosome density (Figure S3B). These analyses of the primary profiling data confirm the conclusions drawn from our simulations of translation: the apparent 5′ ribosome ramp does not actually require a higher density of ribosomes near the 5′ end of each message, but rather it can be explained simply by a greater density of ribosomes on shorter mRNA molecules.

Mapping Ribosome Profile Reads to Genes

The ribosome profiling reads of Ingolia et al. (2009) and their alignment files were downloaded from GEO under the accession number GSE13750. We compared the mapped positions of the sequencing reads to the *S. cerevisiae* genome annotation file downloaded from UCSC genome browser ([Karolchik et al., 2003; Dreszer et al., 2012], genome version June 2008 [SGD/sacCer2]). For each coding sequence, we counted the number of reads that were mapped to each codon (we assigned the read to the codon that mapped to its 17th base), as well as the total number of reads mapped to the sequence. To avoid ambiguity we excluded the reads that were mapped to multiple positions across the genome.

Comparison with Ribosome Flow Model of Translation

The ribosome flow model (Reuveni et al., 2011) describes the translation of an individual mRNA molecule with a fixed rate of initiation and a fixed rate of elongation per codon. By assuming fixed rates of initiation and elongation, the model implicitly assumes a constant, inexhaustible supply of free ribosomes and free tRNAs in the cell. The ribosome flow model describes the translation of each mRNA molecule independently of all other mRNAs, and so the model does not account for competition among mRNAs for free ribosomes or free tRNAs in the cell. In other words, if one mRNA species is highly abundant and densely packed with ribosomes, then this does not limit the pool of available ribosomes to initiate other mRNAs, according to the assumptions of the ribosome flow model. Furthermore, the model predicts that each mRNA is translated close to (93% of) its maximum translation rate (Reuveni et al., 2011; Tuller et al., 2011). As a result, protein translation is generally elongation-limited in the ribosome flow model. This model, which rests on the implicit assumption that free ribosomes are always available, is expected to provide an accurate description of translation in a cell only under conditions in which a very large number of ribosomes are, indeed, free.

To make this point explicit, we have calculated the predicted number of ribosomes bound to mRNAs in a yeast cell based on the estimates of average ribosome density obtained under the ribosome flow model (Reuveni et al., 2011). According to the ribosome flow model, the average ribosome density, per 15 codons, ranges from 0.36 to 0.42 for low-expression and high expression genes, respectively (Reuveni et al., 2011). Therefore, assuming an average ribosome density of 0.4 and a total transcriptome size of 2×10^7 codons (Zenklusen et al., 2008; Ingolia et al., 2009), the number of bound ribosomes predicted by the ribosome flow model is $2\times10^7\times0.4/15=5.33\times10^5$. This number greatly exceeds the total number of ribosomes (free or bound) that have been measured in a real yeast cell (2×10^5 [Warner, 1999; von der Haar, 2008]). As this calculation suggests, the assumptions of the ribosome flow model imply that an unrealistically large number of ribosomes are required to translate all the mRNAs in a yeast cell.

In order to compare the ribosome flow model with our whole-cell simulation of translation, we artificially increased the number of ribosomes and tRNAs in our simulations beyond their empirically measured abundances, so that a large supply of them would be free in equilibrium – in accordance with the assumptions of the ribosome flow model. To do so, we increased the number of tRNA molecules 10-fold (chosen so that there would be a large supply of free tRNAs of all species, even in the extreme case of every ribosome bound to a codon in the transcriptome). We also increased the numbers of ribosomes in the cell, ranging from a 2-fold to a 35-fold increase. To find the regime that corresponds to the ribosome flow model we identified the number of ribosomes required so that protein production in the cell is 93% of its maximal capacity (Figure S5A). To achieve this regime requires a 5-fold increase in the number of simulated ribosomes compared to the true, measured number of ribosomes in a yeast cell. In this regime our simulations recover the elongation-limited behavior of the ribosome flow model – but the total number of ribosomes bound to all mRNAs in the resulting simulated cell is about 8×10^5 (Figure S5A) in this regime, which again exceeds the measured number of ribosomes in a real yeast cell (2×10^5 [Warner, 1999; von der Haar, 2008]), by four-fold. Thus, it is possible for our model to recapitulate the behavior of the ribosome flow model – in which ribosomes are inexhaustibly abundant and the translation dynamics of each mRNA can be treated independently – but to do so requires assuming an unrealistic number of cellular ribosomes.

In summary, the number of ribosomes required to reconcile our cellular model of translation with the ribosome flow model vastly exceeds the number of ribosomes in a normal yeast cell. Likewise, the number of bound ribosomes in the cell, according to direct estimates of ribosome densities inferred by the ribosome flow model (Reuveni et al., 2011), also exceeds the total number of ribosomes measured in a yeast cell. These calculations suggest that the elongation-limited regime described by the ribosome flow model is not realistic for most endogenous genes in a healthy yeast cell.

SUPPLEMENTAL REFERENCES

Curran, J.F., and Yarus, M. (1989). Rates of aminoacyl-tRNA selection at 29 sense codons in vivo. J. Mol. Biol. *209*, 65–77.

Dreszer, T.R., Karolchik, D., Zweig, A.S., Hinrichs, A.S., Raney, B.J., Kuhn, R.M., Meyer, L.R., Wong, M., Sloan, C.A., Rosenbloom, K.R., et al. (2012). The UCSC Genome Browser database: extensions and updates 2011. Nucleic Acids Res. *40*(Database issue), D918–D923.

Fluitt, A., Pienaar, E., and Viljoen, H.J. (2007). Ribosome kinetics and aa-tRNA competition determine rate and fidelity of peptide synthesis. Comput. Biol. Chem. *31*, 335–346.

Gillespie, D. (1977). Exact stochastic simulation of coupled chemical reactions. J. Phys. Chem. *81*, 2340–2361.

Gromadski, K.B., and Rodnina, M.V. (2004). Kinetic determinants of high-fidelity tRNA discrimination on the ribosome. Mol. Cell *13*, 191–200.

Jakubowski, H., and Goldman, E. (1984). Quantities of individual aminoacyl-tRNA families and their turnover in Escherichia coli. J. Bacteriol. *158*, 769–776.

Jorgensen, P., Nishikawa, J.L., Breitkreutz, B.-J., and Tyers, M. (2002). Systematic identification of pathways that couple cell growth and division in yeast. Science *297*, 395–400.

Karolchik, D., Baertsch, R., Diekhans, M., Furey, T.S., Hinrichs, A., Lu, Y.T., Roskin, K.M., Schwartz, M., Sugnet, C.W., Thomas, D.J., et al.; University of California Santa Cruz. (2003). The UCSC Genome Browser Database. Nucleic Acids Res. *31*, 51–54.

Lim, V.I., and Curran, J.F. (2001). Analysis of codon:anticodon interactions within the ribosome provides new insights into codon reading and the genetic code structure. RNA *7*, 942–957.

Nissen, P., Thirup, S., Kjeldgaard, M., and Nyborg, J. (1999). The crystal structure of Cys-tRNACys-EF-Tu-GDPNP reveals general and specific features in the ternary complex and in tRNA. Structure *7*, 143–156.

Tuller, T., Carmi, A., Vestsigian, K., Navon, S., Dorfan, Y., Zaborske, J., Pan, T., Dahan, O., Furman, I., and Pilpel, Y. (2010). An evolutionarily conserved mechanism for controlling the efficiency of protein translation. Cell *141*, 344–354.

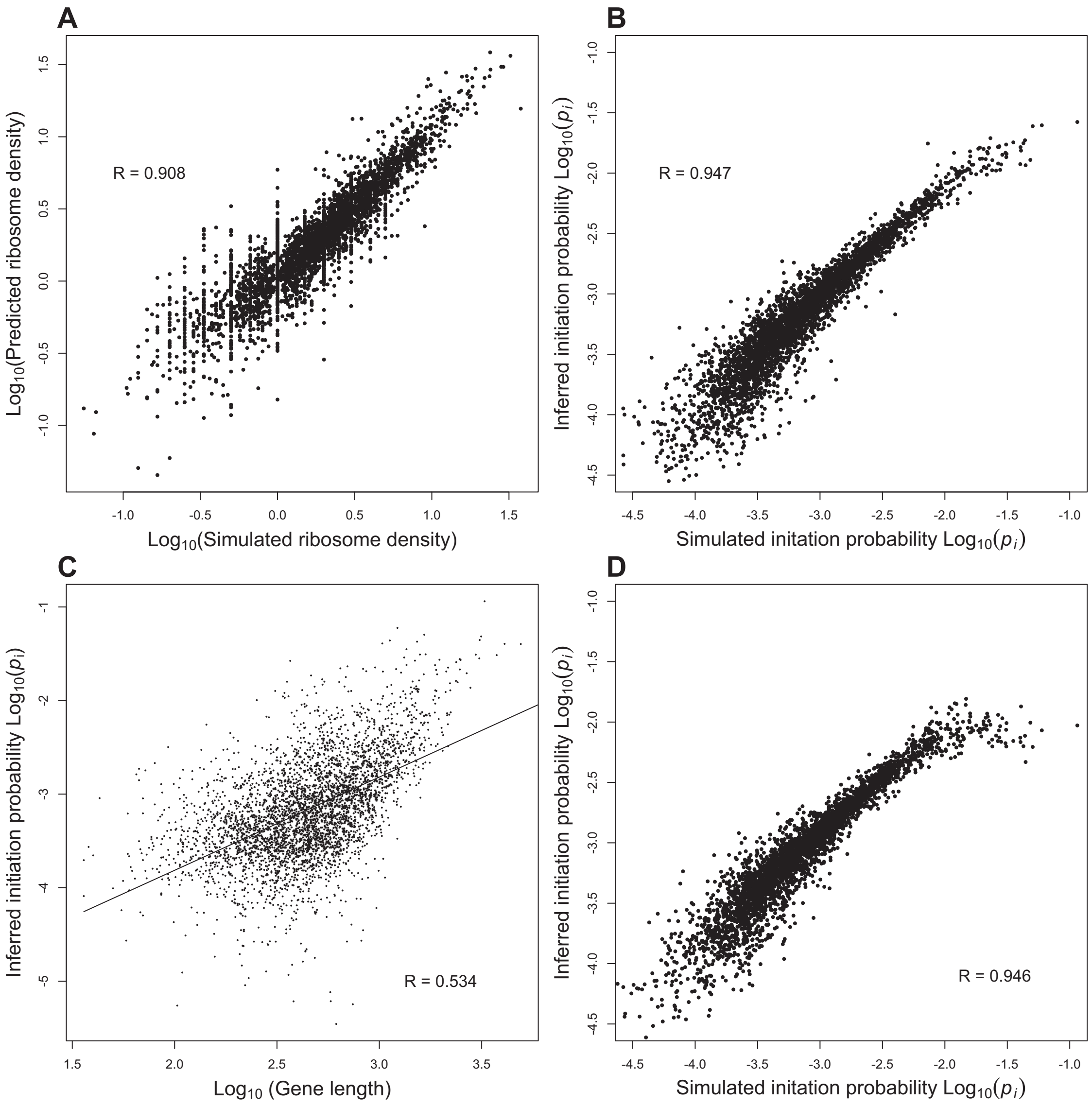

Figure S1. Validation of Analytical Approximations of Simulations Model, Related to Figure 1

(A) We have derived analytic approximations for the steady-state density of ribosomes on each mRNA molecule. These equations provide a good approximation to the true behavior of our stochastic simulation.

(B) We can invert these equations to infer the initiation probability of each gene, based on its observed equilibrium ribosome density. Panel B shows a validation experiment in which we applied this inference method to data that had been simulated with known initiation probabilities, confirming that we can accurately infer these parameters.

(C) Correlation between gene length and initiation probabilities does not bias our inference of initiation probabilities. We validated that we can reliably infer initiation probabilities from observed ribosome densities, even when gene length and initiation probabilities are positively correlated. To do so we inverted the rank order of initiation probabilities assigned to yeast genes, so that the genes with longer ORFs were now assigned greater initiation probabilities.

(D) When we simulated a cell with this new assignment of initiation probabilities we were still able to reliably infer the simulated initiation probabilities based on the simulated ribosome density for each gene. These results confirm that the negative correlation between gene length and inferred initiation probability observed in the real data (main text Figure 5B) is not an artifact of our inference procedure.

Cell

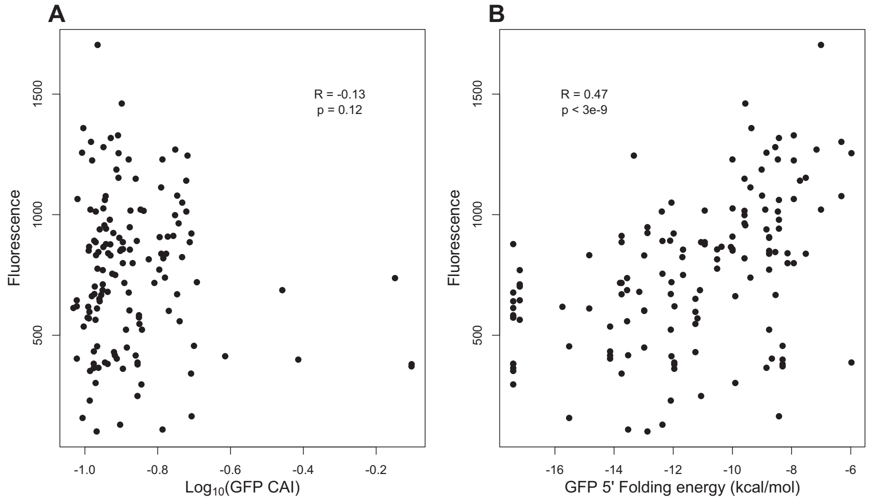

Figure S2. Experimental Validation of Roles of Initiation and Elongation Rates on Protein Production in Yeast, Related to Figures 2 and 4

(A and B) The relationship between protein production, codon bias, and 5′ mRNA folding for 141 synthetic GFP genes expressed in *Saccharomyces cerevisiae*. We constructed 141 GFP genes, all encoding the same protein sequence but varying randomly at synonymous sites, as previously described (Kudla et al., 2009). BY4741 yeast was transformed with a Gal-induced 2-micron plasmid, then grown for 24h in 2% Raf +2% Gal. Fluorescence was then measures by FACS. Measurements typically represent three biological replicates, on the same day. Panel (A) shows the relationship between fluorescence and each GFP gene's yeast Codon Adaptation Index; Panel (B) shows the relationship between fluorescence and each GFP gene's predicted 5′ mRNA folding. 5′ mRNA folding was estimated using mfold, as previously described (Kudla et al., 2009). There is no significant correlation between expression level and codon bias, suggesting that elongation rates do not modulate protein production in these constructs. By contrast, there is a significant correlation between expression level and mRNA folding near the translation initiation site, indicating that initiation rates strongly influence protein production in these constructs.

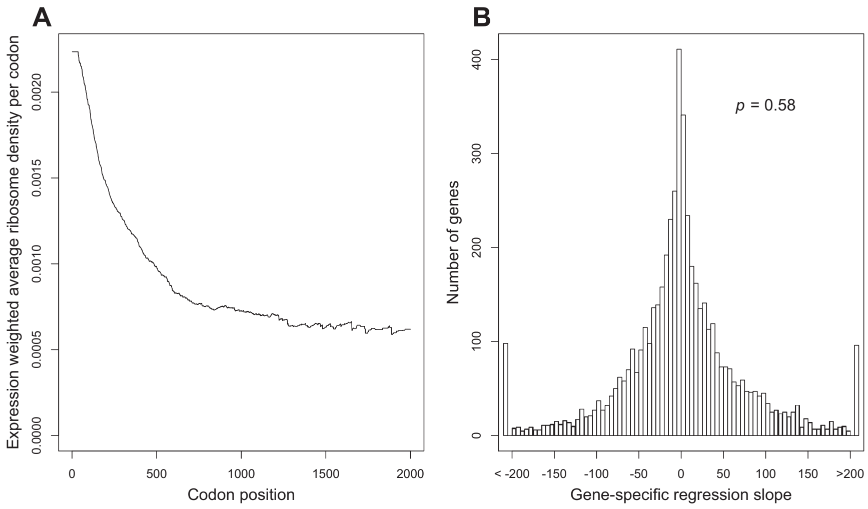

Figure S3. Evidence that the Ramp of 5′ Ribosomes in Profiling Data Is Not Caused by Codon Ordering, Related to Figure 5

(A) We removed all positional information in the profiling data, and used instead only the average ribosome density along each transcript, as reported by Ingolia et al. (2009). When we assume that ribosomes are uniformly distributed along each message, then the figure shows the resulting density of ribosomes as a function of codon position, averaged across the expression-weighted transcriptome. Similar results were obtained using the unweighted transcriptome (data not shown). In both cases we observe a ramp of higher 5′ ribosome density. These results demonstrate that the ramp can be attributed primarily to a greater overall density of ribosomes on shorter genes, even if ribosomes are distributed uniformly along each message.

(B) Evidence that individual genes in yeast do not exhibit a trend toward a 5′ ramp of ribosomes. We analyzed the raw profiling data of Ingolia et al. (2009) and computed, for each yeast gene, the regression between codon position and average ribosome density at that position. A gene with a ramp of elevated 5′ ribosome densities would exhibit a negative slope. Instead, we found that just as many genes exhibit a positive slope as exhibit a negative slope. The mean slope among genes is not significantly different from zero ($p = 0.58$). Thus, there is no systematic trend toward elevated 5′ ribosome densities on individual genes, in the data of Ingolia et al. (2009).

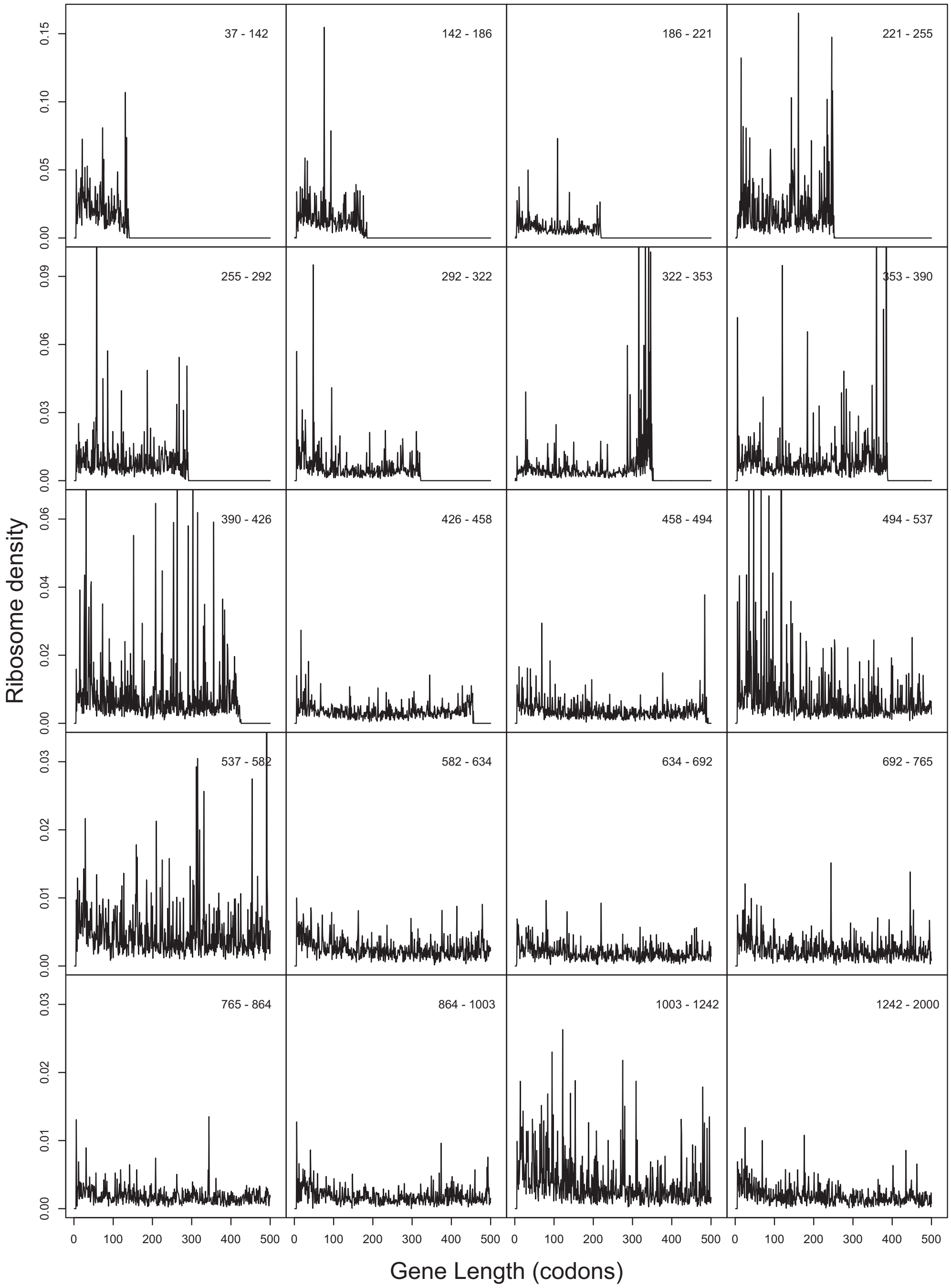

Figure S4. Evidence that Genes Binned by Lengths Do Not Exhibit a Trend toward a 5′ Ramp of Ribosomes, Related to Figure 5

We analyzed the raw profiling data of Ingolia et al. (2009), and computed position-specific ribosome density for groups of genes binned by their ORF lengths. This figures is analogous to Figure S11 of Ingolia et al. (2009), but with more stringent bins on length: each panel contains 5% of genes in the Ingolia data set, sorted according to their lengths. We find no consistent 5′-to-3′ ramp (some panels show 3′-to 5′-ramps) in the data of Ingolia et al. (2009).

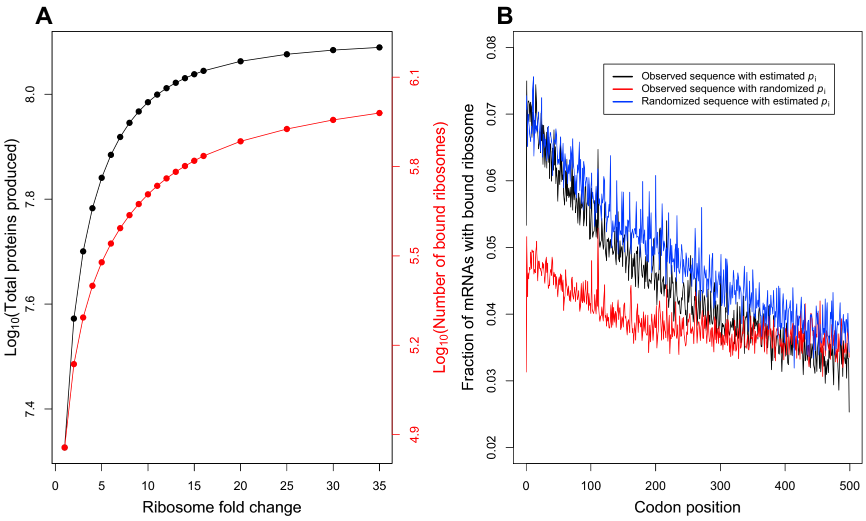

Figure S5. Comparison between Initiation-Limited and Elongation-Limited Regimes of Protein Translation, Related to Figure 5

We simulated our model of translation in a wild-type yeast cell with either the standard number of ribosomes and tRNAs, as used in all other simulations in our study, or alternatively with 10-fold as many tRNAs and an elevated number ribosomes.

(A) We ran multiple simulations that varied the scaling coefficient applied to the number of ribosomes, from 1-fold to 35-fold more than their empirically measured abundance. The number of ribosomes (or, more precisely, the number of free ribosomes) determines the initiation rate of any mRNA whose 5′ end is not currently obstructed by a bound ribosome; and so this quantity corresponds to the initiation rate λ in the ribosome-flow model (Reuveni et al., 2011). Black lines show the total rate of protein production, as a function of the fold-increase in ribosome abundance. As the number of ribosomes is increased, our simulation model behaves like the ribosome-flow model, in which free ribosomes are always abundant and each mRNA is initiated at a fixed rate independent of how many ribosomes are being used to translate other mRNAs; in this regime protein production is elongation-limited. However, the number of ribosomes required to achieve this regime is unrealistic: many more ribosomes are bound to mRNAs (red curve) in this regime than the total number of ribosomes in a real yeast cell.

(B) Simulations under elongation-limited regime of ribosome flow models do produce a “ramp” of decreasing ribosome densities with codon position. However, this decrease persists regardless of codon ordering within genes and to a lesser degree with randomized gene-specific initiation probabilities. This indicates that the ramp produced under the assumptions of ribosome-flow model is an artifact of the underlying elongation-limiting assumption.

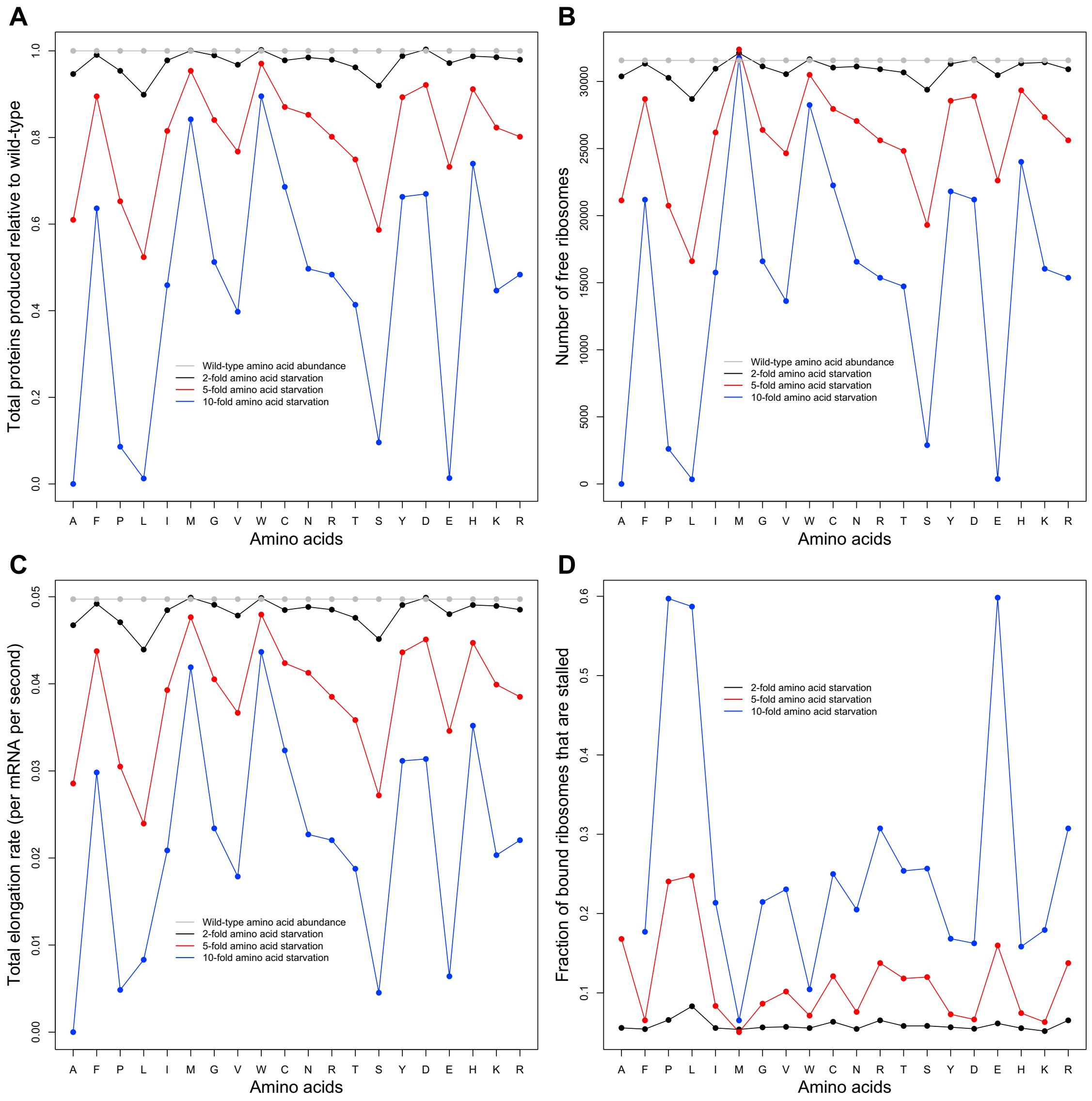

Figure S6. Effects of Amino Acid Starvation on Protein Production, Number of Free Ribosomes, Overall Elongation Rates, and Ribosomal Stalling, Related to Figure 7

All quantities are shown relative to a wild-type cell without any amino acid starvation. We simulated starvation of a particular amino acid by reducing the abundance of all its (charged) cognate tRNAs by either two-, five-, or ten-fold. The rate of protein production decreases under stress, and it can decrease extensively when starvation is severe. Starvation of different amino acids has radically different effects on protein production.

(A–D) Decreased protein synthesis upon starvation is caused primarily by a corresponding decrease in the pool of free ribosomes (comparing panel A to panel B). Starving a cell of amino acids reduces the number of free ribosomes, and this in turn leads to fewer free tRNAs. The combined effects of these two processes lead to an overall decrease in (C) total elongation rates across all mRNAs. Starving a cell of amino acids leads to higher densities of ribosomes bound to mRNAs (D). As a result, greater proportions of bound ribosomes are stalled - that is, they are obstructed from further elongation due to a neighboring ribosome bound 10 codons ahead on the same mRNA.

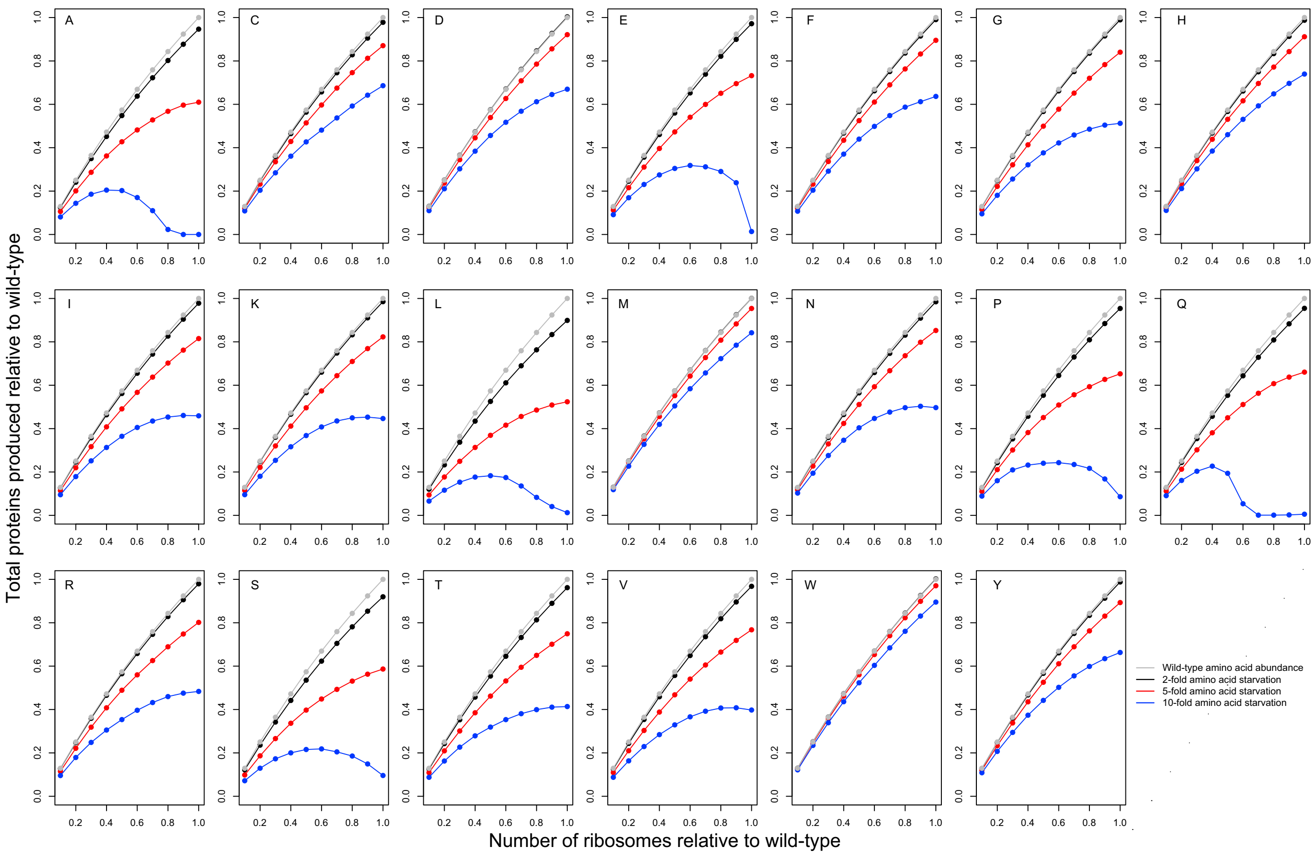

Figure S7. The Effects of Repressing Ribosome Synthesis on Protein Production during Amino Acid Starvation, Related to Figure 7
During starvation, cells usually respond by repressing ribosome production. Reducing ribosomes under normal or mild-stress conditions always reduces protein production. However, for many amino acids (e.g., A, E, L, P, Q, S, V), reducing ribosomes by small amounts partly rescues protein production under amino-acid starvation. The *y* axis shows the total rate of protein production relative to a wild-type cell without any amino-acid starvation.
